# Regulation of Neurogenesis by FGF Signaling and Neurogenin in the Invertebrate Chordate *Ciona*

**DOI:** 10.3389/fcell.2020.00477

**Published:** 2020-06-23

**Authors:** Kwantae Kim, Susanne Gibboney, Florian Razy-Krajka, Elijah K. Lowe, Wei Wang, Alberto Stolfi

**Affiliations:** ^1^School of Biological Sciences, Georgia Institute of Technology, Atlanta, GA, United States; ^2^Department of Biology, New York University, New York, NY, United States

**Keywords:** FGF signaling, Neurogenin, neurogenesis, bipolar tail neurons, *Ciona*, tunicates

## Abstract

Neurogenesis is a complex sequence of cellular processes and behaviors driven by the coordinated expression of conserved effectors. The bipolar tail neurons (BTNs) of *Ciona* develop according to a highly dynamic, yet highly stereotyped developmental program and thus could serve as an accessible model system for neurogenesis, including underlying cell behaviors like neuronal delamination, migration, and polarized axon outgrowth. Here we investigate both the upstream events that shape BTN neurogenesis through spatiotemporal regulation of the conserved proneural factor Neurog, spatiotemporal, and the gene expression profile of differentiating BTNs downstream of Neurog activity. We show that, although early FGF signaling is required for *Neurog* expression and BTN specification, *Fgf8/17/18* is expressed in tail tip cells at later stages and suppresses sustained *Neurog* expression in the anterior BTN (aBTN) lineage, such that only one cell (the one furthest from the source of Fgf8/17/18) maintains *Neurog* expression and becomes a neuron. Curiously, *Fgf8/17/18* might not affect neurogenesis of the posterior BTNs (pBTNs), which are in direct contact with the *Fgf8/17/18-*expressing cells. Finally, to profile gene expression associated with BTN neurogenesis we performed RNAseq of isolated BTN lineage cells in which BTN neurogenesis was enhanced or suppressed by perturbing Neurog function. This allowed us to identify several candidate genes that might play conserved roles in neurogenesis and neuronal migration in other animals, including mammals.

## Introduction

**FIGURE 1:**
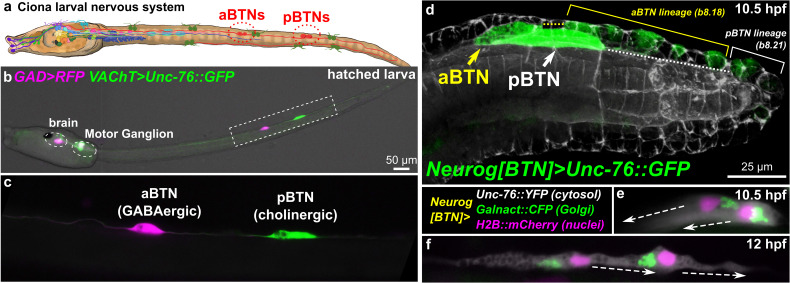
Ciona Bipolar Tail Neurons and the larval nervous system. **(a)** Cartoon diagram of Ciona larval nervous system based on (Ryan et al., 2016), showing approximate positions of posterior BTNs (pBTN), and anterior BTNs (aBTNs). **(b)** *GAD* > *RFP* (Zega et al., 2008) and *VAChT* > *Unc-76:GFP* (Kratsios et al., 2012) reporters label GABAergic aBTNs and cholinergic pBTNs, respectively. Note that due to mosaic incorporation of the reporter plasmids in this particular individual, *VAChT* > *Unc-76:GFP* is not expressed in the cholinergic neurons of the core Motor Ganglion, whose axons normally would obscure the BTNs. **(c)** Magnified view of neurons boxed in **(b)**. **(d)** Confocal image of migrating BTNs in tail tip of a tailbud (11.5 hpf at 18°C, equivalent to ∼10.5 hpf at 20°C) embryo electroporated with *Neurog[BTN]* > *Unc-76:GFP* (green). **(e)** Relative position of Golgi apparatus is posterior to the nucleus in the BTNs during their migration forward (∼11.5 hpf at 18°C or 10.5 hpf at 20°C), then **(f)** becomes anterior to each nucleus during distal process extension (∼13.5 hpf at 18°C or 12 hpf at 20°C). Larva diagram illustration by Lindsey Leigh.

In spite of an emerging picture of the molecular mechanisms of cell fate specification and morphogenesis in neurodevelopment, it is not well understood how these pathways are regulated in different developmental contexts. The simple embryos of the invertebrate chordate *Ciona* are tractable for high-resolution functional genomics (Reeves et al., 2017; Horie et al., 2018; Racioppi et al., 2019; Wang et al., 2019) and *in vivo* imaging (Cota and Davidson, 2015; Hashimoto et al., 2015; Veeman and Reeves, 2015; Mizotani et al., 2018; Bernadskaya et al., 2019), and have been increasingly used to investigate the regulation of cell behaviors in development (Bernadskaya and Christiaen, 2016). Furthermore, their classification in the tunicates, the sister group to the vertebrates (Delsuc et al., 2006), means they share with vertebrates many chordate-specific gene families, cell types, organs, and developmental processes (Ermak, 1977; Ogasawara and Satoh, 1998; Christiaen et al., 2002; Hervé et al., 2005; Dufour et al., 2006; Kugler et al., 2008; Stolfi et al., 2010, 2011, 2015; Razy-Krajka et al., 2012; Tolkin and Christiaen, 2012; Abitua et al., 2015), particularly their larval central nervous system (CNS), a miniaturized but typically chordate CNS containing only 177 neurons (Figure 1a; Ryan et al., 2016). *Ciona* are thus model organisms well-suited to the study of potentially conserved, chordate-specific gene regulatory networks controlling neurogenesis and associated cell behaviors during neurodevelopment.

To study these processes in *Ciona* neurons, we have focused on the Bipolar Tail Neurons (BTNs, Figures 1b,c). The BTNs are two bilateral pairs of neurons located along the tail nerve cord and derive their name from the two long processes they extend in opposite directions along the anterior-posterior axis. Each left/right pair is comprised of a GABAergic anterior BTN (aBTN) and a cholinergic posterior BTN (pBTN) that arise from separate but adjacent lineages (Figure 1d). The BTNs are proposed homologs of vertebrate dorsal root ganglia (DRG) neurons, based on their developmental origin from the neural crest-like cells, their early expression of Neurogenin (Neurog) family of proneural transcription factors, their morphogenesis, and their role in relaying peripheral sensory information to the CNS (Stolfi et al., 2015). Like neural crest-derived DRG neurons in vertebrates, BTNs delaminate from the dorsal midline ectoderm and migrate along paraxial mesoderm as a simple chain comprised of the aBTN followed by the pBTN (Figure 1d), achieving their unique morphology by first extending a neurite anteriorly (Figure 1e), then reversing polarity and extending a neurite posteriorly (Figure 1f).

It was previously shown that FGF/ERK signaling regulates BTN lineage specification and cell fate choice (Stolfi et al., 2015). Early treatment (5 h post-fertilization, or hpf, at 20°C, equivalent to the St.12 mid-gastrula stage) with the MEK inhibitor U0126 abolished Neurog expression and BTN specification. In contrast, later treatment (7 hpf 20°C, St.16 late neurula) with U0126 paradoxically resulted in ectopic, sustained Neurog expression within the BTN lineage, resulting in the specification of supernumerary BTNs at the expense of other cells in the lineage. The roles of other signaling pathways in specifying BTN fate are not yet clear. For instance, Delta/Notch perturbation does not appear to affect BTN specification or differentiation (Stolfi et al., 2015).

The dynamic, opposing roles of FGF/ERK signaling in controlling BTN specification and differentiation is consistent with several observations on the paradoxical roles of FGF in regulating vertebrate neurogenesis (Diez del Corral and Morales, 2017), as well as other tissues in *Ciona*, for instance the heart (Davidson et al., 2006; Razy-Krajka et al., 2018). For instance, early FGF signaling is required for specification of neuromesodermal precursors (Storey et al., 1998; Boulet and Capecchi, 2012; Sasai et al., 2014). However, sustained FGF signaling in these cells later promotes a mesoderm fate over neuronal fate (Boulet and Capecchi, 2012; Henrique et al., 2015), as it does in the *Ciona* neuromesodermal “A9.32” blastomeres that give rise to motor neurons and paraxial tail muscles (Hudson et al., 2007; Navarrete and Levine, 2016). Similarly, FGF signaling is required for neural crest specification (Sasai et al., 2014), but sustained FGF signaling in the dorsal neural tube keeps cells in an uncommitted, non-neural crest state (Martínez-Morales et al., 2011). Thus, the regulated downregulation of FGF signaling in these cells promotes delamination and migration of neural crest cells, including those that will give rise to DRG neurons (Martínez-Morales et al., 2011). Finally, downregulation of FGF signaling has been shown to be crucial for mitotic exit and neuronal differentiation in both vertebrates (Diez del Corral et al., 2002) and *Ciona* (Stolfi et al., 2011).

It was also previously shown that sustained expression of Neurog is necessary and sufficient for BTN specification, delamination, and migration, as supernumerary BTNs generated by ectopic Neurog overexpression engage in these same stereotyped behaviors (Stolfi et al., 2015). In vertebrates, Neurog2 is activated in delaminating mammalian neural crest cells, long before commitment to a neuronal fate (Soldatov et al., 2019). Neurog1/Neurog2 are also expressed in committed DRG progenitors as they migrate through somatic mesoderm and begin to differentiate into their bipolar (more accurately pseudounipolar) shape to transmit sensory information from peripheral tissues to the CNS (Ma et al., 1999). Therefore, Neurog factors might be activating conserved regulatory “programs” for migration, polarization, and axon outgrowth of neural plate border-derived sensory neurons that are shared between tunicates and vertebrates. Since Neurog family factors are expressed in many other differentiating neurons throughout the CNS, it is thought that many of their direct and indirect transcriptional targets might also be shared among various different neuron types and conserved throughout metazoan evolution. However, these targets have not been profiled in detailin migrating sensory neuron precursors.

In this study, we investigated the role of FGF signaling in regulating *Neurog* expression and subsequent BTN neurogenesis. Although it has been shown that Fgf9/16/20 is required to specify neural plate border cells (Roure et al., 2014), from which both aBTN and pBTN lineages arise, here we demonstrate that later Fgf8/17/18 from tail tip cells controls neural differentiation in the aBTN (but not pBTN) lineage. More specifically, we show that tail-tip Fgf8/17/18 is required to suppress sustained Neurog expression in the majority of the aBTN lineage-derived cells, resulting in the eventual differentiation of only two BTNs per side. However, pBTNs appear unaffected by manipulating either Fgf8/17/18 function or inhibiting FGF signaling in general.

Additionally, we use RNAseq to profile migrating BTNs under Neurog gain- or loss-of-function conditions, dissociated, and isolated from synchronized embryos using fluorescence-activated cell sorting (FACS). By analyzing BTN transcriptome profiles under these conditions, we identified, and validated by *in situ* hybridization, a core set of candidate “effector” genes downstream of BTN fate choice, many of them highly conserved in vertebrate neurogenesis. This and other genes encode a diverse set of intracellular and extracellular proteins that provide an entry point to studying the molecular pathways that control BTN neurogenesis, delamination, migration, and morphogenesis. Thus, our work in characterizing gene regulatory mechanisms acting both upstream and downstream of the critical determinant of BTN fate, Neurog, sets a foundation for the dissection of a potentially conserved, and chordate-specific transcriptional network for morphogenetic cell behaviors in neurogenesis.

## Results and Discussion

### Distinct FGFs Control BTN Lineage Specification and Cell Fate Decisions

Because treatment with the MEK inhibitor U0126 has opposing effects on BTN specification depending on timing (Stolfi et al., 2015), we reasoned that different FGF signaling events might be controlling (1) initial *Neurog* expression and BTN lineage specification between 5 and 7 hpf and (2) later restriction of *Neurog* within the BTN lineage, after 7 hpf. *Fgf9/16/20* is the earliest *Fgf* family gene expressed (starting at the 16-cell stage onwards) and has been previously shown to be required for the specification of the posterior neural plate borders and for the activation of the conserved neural plate border regulatory gene *Msx* (Roure et al., 2014). *Msx* in turn has been shown to be required for BTN specification and differentiation (Li et al., 2017). Therefore, Fgf9/16/20 signaling is required for initial BTN lineage specification, which is consistent with the complete loss of *Neurog* expression upon early U0126 treatment. However, this activating function is at odds with the later effect of U0126 treatment, which results in ectopic *Neurog* expression and supernumerary BTNs instead. We therefore sought to understand more clearly how this later FGF signaling component might function.

**FIGURE 2:**
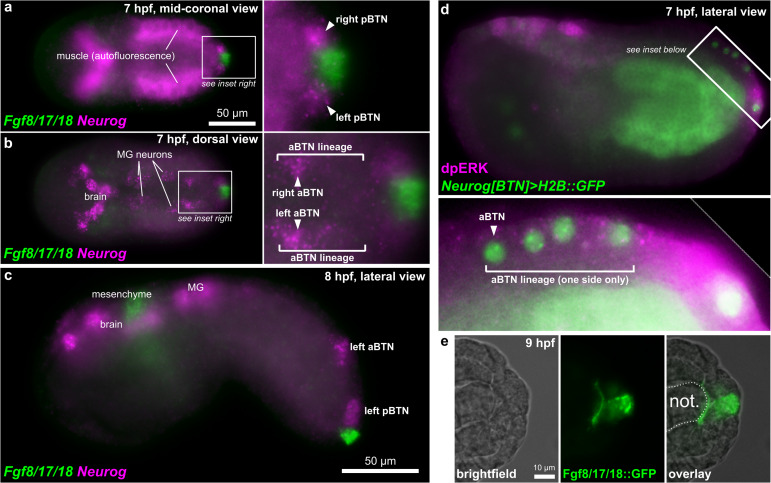
BTNs and the FGF signaling pathway. **(a)** Two-color *in situ* hybridization at 7 hpf showing *Fgf8/17/18* and *Neurog* expression in pBTN lineage (magnified inset). **(b)** Same embryo as in **(a)**, but viewed at a more dorsal focal plane, showing *Neurog* expression in the aBTN lineage and relative position of the aBTNs and *Fgf8/17/18* expression in the tail tip (magnified inset). **(c)** Two-color *in situ* hybridization at 8 hpf showing migrating aBTN and pBTN cells on one side of the embryo. **(d)** Immunohistochemical staining for phosphorylated ERK (dpERK, magenta) in a 7 hpf embryo, showing posterior-to-anterior gradient in the aBTN lineage (magnified inset). aBTN lineage is labeled with *Neurog[BTN]* > *H2B:GFP* reporter plasmid expression (green nuclei). **(e)** Embryo electroporated with *Fgf8/17/18* > *Fgf8/17/18:GFP* plasmid, showing Fgf8/17/18:GFP (green) emanating from the tail tip cells, spreading around the tip of the notochord. MG: Motor Ganglion. Not.: Notochord.

**FIGURE 3:**
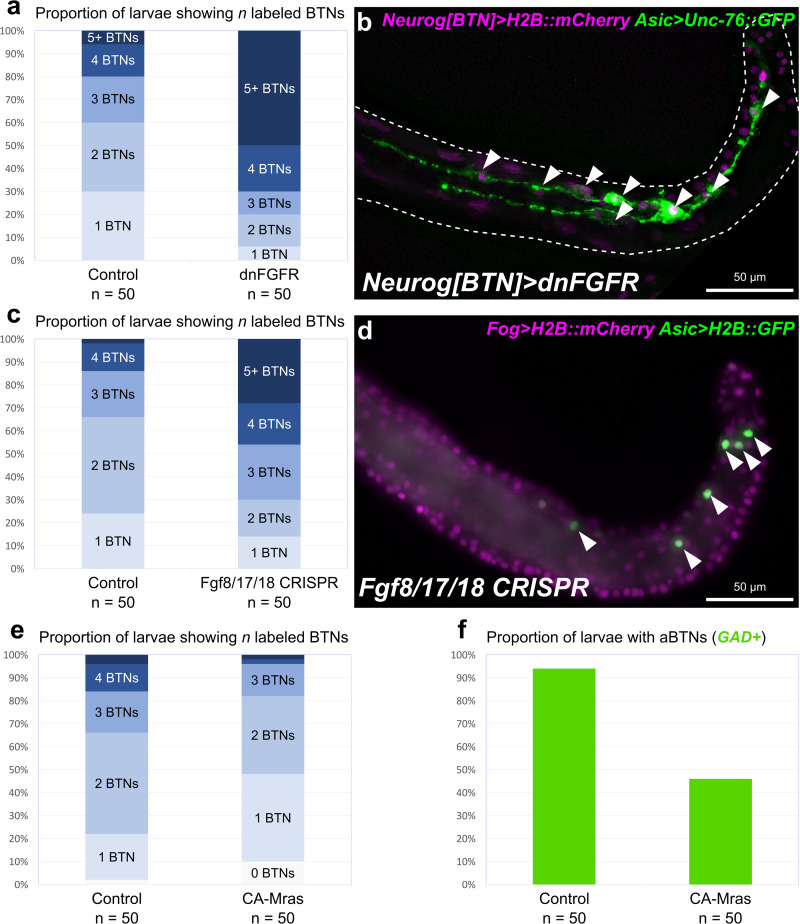
Perturbing FGF signaling in the BTN lineages. **(a)** Quantification of number of *Asic* > *Unc-76:GFP* + BTNs seen in larvae, showing expansion of BTNs upon dnFGFR overexpression. Only larvae with both Unc-76:GFP and BTN lineage-specific *Neurog[BTN]* > *H2B:mCherry* expression were scored. **(b)** Representative image of a larva showing many supernumerary BTNs (arrowheads) upon dnFGFR overexpression in the BTN lineages. **(c)** Quantification of BTN specification as in **(a)** but for tissue-specific CRISPR/Cas9-mediated knockout of *Fgf8/17/18*, using *Asic* > *H2B:GFP* as the BTN fate marker. **(d)** Representative image of a larva showing supernumerary BTN nuclei (arrowheads) upon tail tip-specific knockout of *Fgf8/17/18.* **(e)** Quantification of BTN specification as in (a) using *Asic* > *Unc-76:GFP*, but for overexpression of CA-Mras. In this case, H2B:mCherry + larvae with zero BTNs were also counted. **(f)** Quantification of larvae with *GAD* > *Unc-76:GFP-*labeled aBTNs upon CA-Mras overexpression.

Starting at 7 hpf, a different *Fgf* family gene, *Fgf8/17/18* is expressed in tail tip cells adjacent to the pBTNs (Figure 2a). According to our previous work on BTN lineage studies, these *Fgf8/17/18* + cells are likely derived from the same immediate lineage as the pBTNs (Stolfi et al., 2015). At this moment, *Neurog* expression has become restricted to the anteriormost cell in the aBTN lineage on either side of the midline, furthest from the tail tip, the source of Fgf8/17/18 (Figures 2b,c). The expression of *Fgf8/17/18* in tail tip cells that are touching the pBTNs suggested that FGF signaling might not have a negative effect on *Neurog* expression in these cells. However, the tail tip localization of *Fgf8/17/18* is more consistent with a role for restricting aBTN fate, through a posterior-to-anterior concentration gradient. To assay FGF signaling levels in the region, we performed dpERK antibody staining at 7 hpf, which revealed a posterior-to-anterior gradient of ERK phosphorylation along the dorsal midline (Figure 2d). We observed highest levels of phosphorylation (and presumably, FGF signaling activity) in more posterior cells closest to the tail tip, and lowest levels in the presumptive aBTN cell which is furthest from the tail tip. FGF signaling in other *Ciona* cell fate decision events has mostly been observed as the result of direct cell-cell contacts (Hudson et al., 2007; Imai et al., 2009; Guignard et al., 2018). We therefore asked whether we could find Fgf8/17/18 localized at a longer distance from its source. When we expressed an Fgf8/17/18:GFP fusion protein in the tail tip under the control of the endogenous *Fgf8/17/18* promoter, we found that most GFP signal was localized to the tail tip cells, but that some was also observed localized around the extracellular matrix between the notochord and the overlying ectoderm (Figure 2e and Supplemental Figure 1). It is unclear whether this represents secreted, extracellular Fgf8/17/18, or if is carried by filopodia, cytonemes, or extra-cellular vesicles, etc. However, this distribution is consistent with the proposed action of Fgf8/17/18 at a distance from the tail tip. Alternatively, it is possible that Fgf8/17/18 acts only over cell-cell contact very early on, with later anterior/posterior differences in dpERK and *Neurog* activity arising through asymmetric propagation of downstream, intracellular signaling as the cells in the lineage divide and proliferate. Either way, *Neurog* expression in the aBTN lineage is inversely correlated with distance from the source of Fgf8/17/18, suggesting a negative effect of late FGF signaling on BTN specification. To test whether FGF signaling is restricting BTN specification, we first expressed a truncated, dominant-negative FGF receptor (Davidson et al., 2006) in BTN lineages using the *Neurog[BTN]* driver (Stolfi et al., 2015; *Neurog* > *dnFGFR*). This resulted in supernumerary BTNs in a substantial proportion of larvae (Figures 3a,b). Using the *Asic* reporter to visualize differentiated BTN fate, a majority (>90%) of embryos had fewer than 4 BTNs labeled in control embryos expressing an inert *lacZ* transgene (*Neurog* > *lacZ*), which is expected due to mosaic uptake of the reporter. However, in the dnFGFR condition, a majority (>70%) had 4 or more BTNs labeled, and half had more than 5 BTNs labeled, clearly indicating an excess number of BTNs. This mimics the previously published U0126 result and further demonstrates a cell-autonomous requirement for FGF signaling in BTN precursors to limit BTN differentiation.

To test whether *Fgf8/17/18* is necessary for BTN fate restriction, we used CRISPR/Cas9 to knock this gene out specifically in the animal-pole derived ectoderm (a-/b-lineages), which gives rise to the tail tip. No other cells derived from these lineages express *Fgf8/17/18* at the tailbud stage, and *Fgf8/17/18* in other cells was not disrupted thanks to the use of *Fog* > *Cas9* to restrict Cas9 expression to the animal pole (Gandhi et al., 2017). In embryos electroporated instead with a non-specific “control” single-chain guide RNA (sgRNA), we detected fewer than 4 BTNs labeled in over 85% of embryos (Figure 3c). In contrast, knocking out *Fgf8/17/18* in the tail-tip resulted in over 40% of embryos with 4 or more BTNs, and over 25% of embryos with more than 5 BTNs (Figures 3c,d). Although the effect was not as pronounced as the dnFGFR overexpression, these data are consistent with a role for Fgf8/17/18 ligand emanating from the tail tip to restrict BTN specification after initial *Neurog* activation has been initiated in the lineage.

**FIGURE 4:**
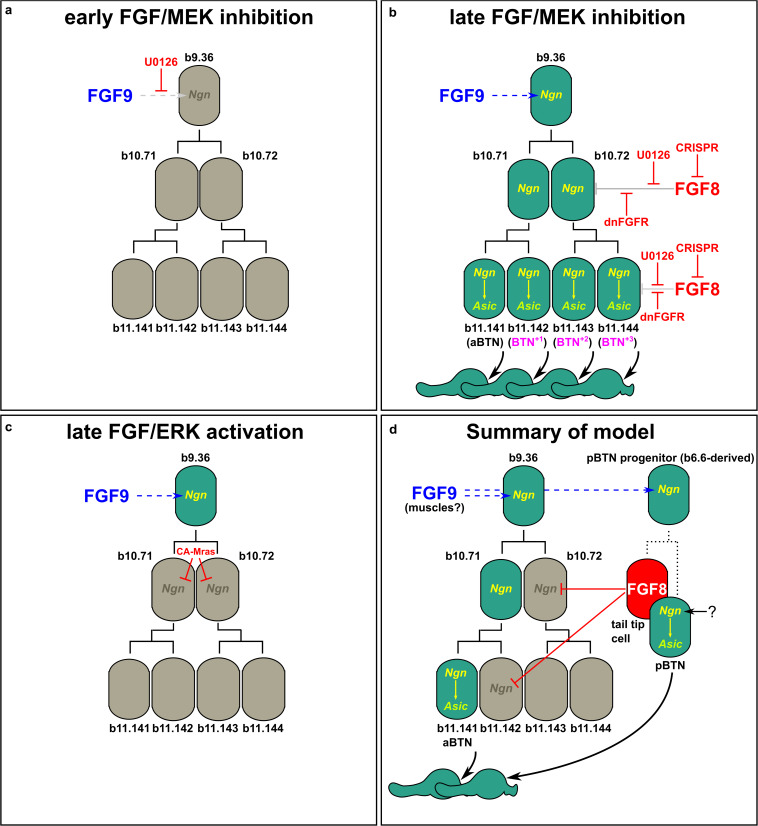
Models of FGF-dependent regulation of BTN specification and differentiation. **(a)** Early U0126 treatment confirms role of Fgf9/16/20 (FGF9) in specifying BTN lineage founder cells (from Stolfi et al., 2015). **(b)** Perturbing late FGF signaling, either via late U0126 treatment (Stolfi et al., 2015), or dnFGFR overexpression or *Fgf8/17/18* knockout using CRISPR results in supernumerary aBTNs, through loss of repression of *Neurog* in posterior cells of the aBTN lineage. **(c)** Ectopic FGF/ERK activation via CA-Mras overexpression suppresses maintenance of *Neurog* expression and abolished aBTN fate. **(d)** Summary of our model encompassing the distinct roles of early and late FGF signals, and the distinct aBTN lineage-specific requirement for *Fgf8/17/18* to restrict differentiation.

To test whether FGF/ERK signaling is sufficient to restrict BTN fate specification, we overexpressed a constitutively active form of Mras (CA-Mras), which transduces FGF signaling upstream of MEK/ERK (Razy-Krajka et al., 2018). We overexpressed CA-Mras in the BTN lineages by electroporating the embryos with *Neurog[BTN]* > *CA-Mras* and assayed its effect on *Asic* reporter plasmid expression. Although there was a reduction in average number of *Asic* > *Unc-76:GFP*-labeled BTNs in CA-Mras-expressing larvae (Figure 3e), there were few larvae that had no visible BTNs at all. We supposed this might be due to the fact that sustained FGF/ERK might restrict only aBTN (but not pBTN) fate specification, as predicted by the *Fgf8/17/18* expression pattern. To further test this hypothesis, we repeated the CA-Mras overexpression while assaying expression of the *Glutamate decarboxylase (GAD)* reporter plasmid (Zega et al., 2008), that labels only the differentiated aBTNs, not pBTNs (see Figure 1). Indeed, CA-Mras overexpression greatly suppressed aBTN differentiation (Figure 3f). Unfortunately, we were unable to use a similar pBTN reporter to assay pBTN specification exclusively. Although the *Vesicular acetylcholine transporter (VAChT)* reporter (Yoshida et al., 2004) is active in the pBTN but not aBTN (Figures 1b,c), it also often expressed in other tail neurons and Motor Ganglion (MG) neuron axons that extend throughout the tail, making pBTN specification very difficult to assay. Therefore, assaying the activity of a more specific pBTN reporter in both gain-of-function CA-Mras and loss-of-function (*Fgf8/17/18* CRISPR) conditions will be needed to fully assess the role of late FGF signaling on this sub-lineage. However, taking the above results together with the direct contact between Fgf8/17/18-expressing tail tip cells and the differentiating pBTNs (Figure 2a), we suggest that Fgf8/17/18 is key for restricting the number of aBTNs, but not pBTNs. We summarize our current model of FGF signaling and BTN neurogenesis using a diagram (Figure 4).

### RNAseq Profiling of Potential Effectors of Neurogenesis in Isolated BTN Progenitors

Because Neurog overexpression is sufficient to specify ectopic differentiated BTNs that all delaminate and migrate (Stolfi et al., 2015), we sought to identify those genes that are upregulated downstream of Neurog, as some may encode effectors of BTN neurogenesis and cell behaviors. Although Neurog is a transcription factor, it is important to note that not all of these effectors are expected to be direct transcriptional targets of Neurog. However, we still consider these to be “downstream” of Neurog.

**FIGURE 5:**
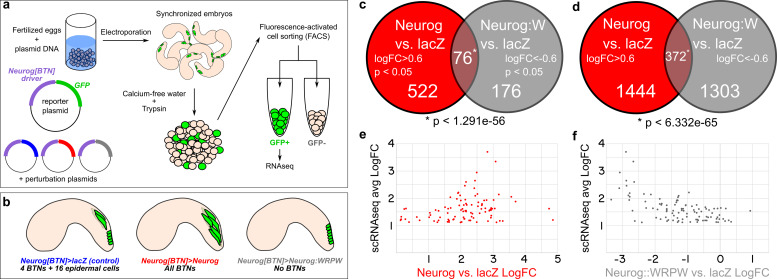
RNAseq-based analyses of Neurog function in BTNs. **(a)** Schematic of FACS + RNAseq approach used to profile BTNs. **(b)** Schematic of different conditions used to sort “control” BTN lineages and Neurog gain- or loss-of-function. **(c)** Non-proportional Venn diagram indicating number of genes in each condition showing statistically-significant (*p* < 0.05) differential expression (LogFC > 0.6 in Neurog vs. lacZ or <-0.6 in Neurog:WRPW vs. lacZ). Statistical significance (asterisk) was calculated using hypergeometric test. **(d)** Same analysis as in **(c)** but with *p*-value cutoff removed. Hypergeometric test was also used to measure statistical significance (asterisk). **(e)** Comparison of “avg LogFC” of top 98 BTN genes identified by single-cell RNAseq (Horie et al., 2018) to LogFC in Neurog vs lacZ, showing that all 98 are positively upregulated by Neurog. **(f)** Similar comparison as in **(e)** but to LogFC in Neurog:WRPW vs. lacZ, revealing that all but 7 of the top 98 BTN genes are downregulated by Neurog:WRPW. “Neurog:W” = Neurog:WRPW.

To identify these direct or indirect downstream genes, we turned to transcriptome profiling using FACS-RNAseq (Figure 5a). We profiled cells labeled with a *Neurog[BTN]* fluorescent reporter under different experimental conditions, isolated from synchronized embryos at 9.5 hs post-fertilization (hpf) at 20°C. In the “control” condition (*Neurog* > *lacZ*) only 4 cells per embryo become BTNs, while the rest of the BTN lineage is initially specified as broadly epidermis (∼15–16 cells at mid-tailbud), with various epidermal sensory neurons specified later (Figure 5b; Stolfi et al., 2015). In parallel, we sorted cells from embryos in which wild-type Neurog was overexpressed (*Neurog* > *Neurog*), or a dominant-repressor form of Neurog (*Neurog* > *Neurog:WRPW*). *Neurog* > *Neurog* specifies all cells as supernumerary BTNs, while *Neurog* > *Neurog:WRPW* abolishes BTN fate (Figure 5b). cDNA libraries were prepared from isolated cells, with each condition represented by two biologically independent replicates.

Under these conditions, 522 genes (of a total of 11,777 analyzed) were upregulated by Neurog (LogFC > 0.6, *p* < 0.05) and 176 downregulated by Neurog:WRPW (LogFC < −0.6, *p* < 0.05), with 76 genes in both categories (*p* < 1.291e-56 using the hypergeometric test, Figure 5c and Supplemental Table 1). The larger number of genes upregulated by Neurog overexpression was expected, given that many more ectopic BTNs are specified in *Neurog* > *Neurog* than the number of BTNs lost in *Neurog* > *Neurog:WRPW* (Stolfi et al., 2015). However, this could also be an artifact due to lower statistical support as a result of vastly different numbers of cells sorted between *Neurog* > *Neurog:WRPW* replicates (2418 cells and 114 cells). Although there were reported whole-mount *in situ* hybridization (ISH) images for 33 of these 76 genes on the ANISEED tunicate expression database (Brozovic et al., 2018), we were able to infer clear BTN expression from such database images for only 10 genes. These included the marker gene *Asic* previously used to assay BTN specification (Coric et al., 2008), and additional genes such as *alpha-Tubulin* (KH.C8.892), *Rgs19/20 (KH.C1.314), Slc35g2 (KH.L141.43), Bassoon-like (KH.C5.481), Onecut*, and others with no substantial homology to known proteins. Because several other known BTN markers were not represented, we relaxed our criteria. More specifically, we looked at genes that were upregulated by Neurog (1444) and downregulated by Neurog:WRPW (1303) with no *p*-value cutoff. This increased the overlapping set, and thus our candidate target gene list, to 372 genes (Figure 5d). This overlap was still statistically significant (*p* < 6.332e–65), suggesting this expanded list is likely to include bona fide BTN-specific genes downstream of Neurog.

To further test whether we were measuring meaningful BTN-specific gene expression, we cross-referenced these data to a previously published single-cell RNAseq data set comprising the top 100 genes enriched in the BTNs relative to other cell types at 12 hpf at 18°C (Horie et al., 2018; Supplemental Table 2), with the exception of two genes: *KH.S1555.2* (which was not present in our dataset), and *Neurog* (due to confounding reads from the electroporated plasmids). We found that all 98 top BTN genes in the scRNAseq dataset were positively regulated by Neurog overexpression (LogFC > 0, Figure 5e). Similarly, 91 of 98 top genes were negatively regulated by Neurog:WRPW overexpression (LogFC < 0, Figure 5f). This confirmed that Neurog positively regulates BTN fate, and that our strategy was able to detect differential gene expression in the BTNs downstream of Neurog activity, though statistical support might be lacking for many BTN markers at the embryonic stages that were sequenced.

### Validation of BTN Genes by *in situ* Hybridization

**FIGURE 6:**
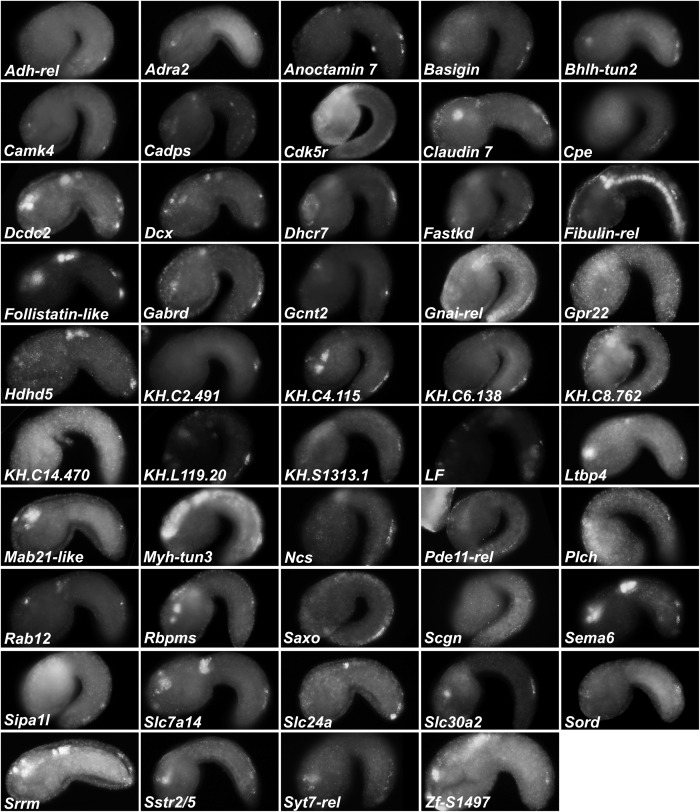
Fluorescent whole-mount *in situ* hybridization of Neurog targets (all 49 validated candidates).

Because the above results suggested our differential expression analysis criteria might (1) be too stringent to detect all real BTN-specific genes downstream of Neurog and (2) might contain false-positives associated with leaky expression of the Neurog driver in other tissues, we decided to validate a large set of potential BTN markers by fluorescent ISH (Supplemental Table 3). We successfully prepared probes for 137 genes, from a mixture of cDNA clones, RT-PCR, and synthetic DNA templates (see section “Materials and Methods” for details, and Supplemental Table 3 for all probe template sequences). Of these, 49 were confirmed to be upregulated in the migrating BTNs (Figure 6). For another 30, it was not clear if they were expressed in BTNs or not, due to low signal or obscuring signal from neighboring tissues. Most are likely true positives, but confirming them will require better probes or higher resolution imaging. 15 genes showed CNS-specific expression, but in other neurons, 15 showed expression mainly in non-neural tissues, and 29 were true “negatives” with no or little signal throughout the whole embryo (all images available at https://osf.io/uqfn2/).

From our results it became obvious that validation of BTN expression by ISH in this subset correlated most closely with overall transcript abundance in the samples. 22 of the top 50 genes with highest LogCPM were BTN+, with another 10 showing “unclear” signal. In contrast, only 3 of the 50 genes with lowest LogCPM were BTN+, though 11 were “unclear.” 23 of the bottom 50 genes were “negative,” suggesting that many of these might in fact be expressed in the BTNs, but at levels that are below the threshold of detection by ISH. Among those genes that were validated by ISH as specifically upregulated in BTNs during delamination and axon extension, some are expressed in either the aBTN, or pBTN alone, though it is unclear if this indicates merely a difference in timing of gene expression between the two. However, there is reason to believe that there are functional differences between the aBTN and pBTN. For instance, the GABAergic marker *GAD* (Zega et al., 2008) is only ever seen to be expressed in the aBTN (Figure 1c), while the cholinergic markers *VAChT/ChAT* (Yoshida et al., 2004) are expressed in the pBTN (Figure 1b). Both are upregulated by Neurog (*GAD* LogFC = 2.7, *Slc18a3/VAChT* LogFC = 3) and downregulated by Neurog:WRPW (*GAD* LogFC = −1.4, *Slc18a3/VAChT* LogFC = −1.1), suggesting that *Neurog* might regulate both targets but in separate aBTN/pBTN contexts.

We also found that many genes were expressed in other CNS neurons where *Neurog* is known to be expressed, in addition to BTNs. Such genes are potentially downstream of Neurog in these other CNS neurons, especially in the MG and brain. Thus, Neurog is likely to directly and indirectly regulate overlapping sets of genes that can be broadly neuronal, BTN-specific, or aBTN/pBTN-specific, highlighting the importance of combinatorial regulation with other lineage-specific transcription factors in regulating neuronal subtype-specific fates and gene expression.

### CRISPR/Cas9-Mediated *Neurog* Loss-of-Function Mutations Abolish BTN Effector Gene Expression

Although Neurog:WRPW was used for our RNAseq profiling due to its robust ability to completely abolish all BTNs in *Ciona*, true *Neurog* loss-of-function in the BTN lineage has not yet been shown. We thus used *Fog* > *Cas9* to target *Neurog* for CRISPR/Cas9-medated mutagenesis specifically in the a/b-lines. We co-electroporated this with a previously published and validated sgRNA targeting *Neurog* (*Neurog.1)*, and two additional validated sgRNAs targeting the proximal promoter of *Neurog* (*Neurog.p1* and *Neurog.p2)*, after attempts to validate other coding sequence-targeting sgRNAs failed. The combined activity of all three sgRNA expression vectors is predicted to frequently result in a large deletion spanning most of the gene, as previously demonstrated in *Ciona* (Gandhi et al., 2017).

**FIGURE 7:**
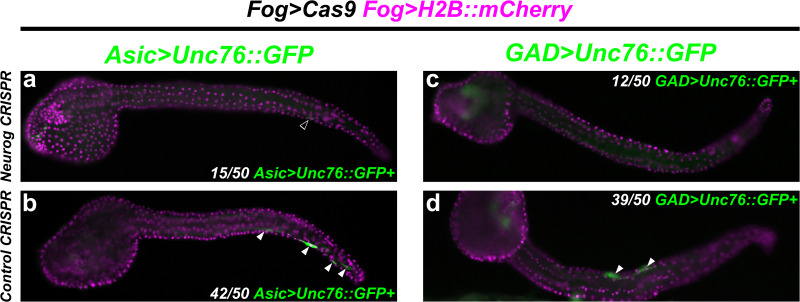
CRISPR/Cas9-mediated knockout of *Neurog.* **(a)** Tissue-specific (using *Fog* > *Cas9)* CRISPR/Cas9-mediated knockout of *Neurog*, using *Asic* > *Unc-76:GFP* as a reporter. Only 15/50 of *Fog* > *H2B:mCherry* + embryos also showed *Asic* > *Unc-76:GFP-*expressing BTNs. Open arrowhead indicates BTN with very faint reporter expression. **(b)** Control CRISPR embryo, showing *Asic* > *Unc-76:GFP-*expressing BTNs (white arrowheads). 42/50 of *Fog* > *H2B:mCherry* + control embryos had *Asic* > *Unc-76:GFP-*expressing BTNs. **(c)** Same conditions as in **(a)** but using *GAD* > *Unc-76:GFP* as an aBTN marker. Only 12/50 *Fog* > *H2B:mCherry* + embryos also showed *GAD* > *Unc-76:GFP-*expressing aBTNs. **(d)** Control CRISPR embryo, showing *GAD* > *Unc-76:GFP-*expressing aBTNs (white arrowheads). 39/50 of *Fog* > *H2B:mCherry* + control embryos had *GAD* > *Unc-76:GFP-*expressing aBTNs.

Indeed, targeting *Neurog* in this way resulted in dramatic loss of *Asic* > *Unc-76:GFP* reporter expression in F0 embryos, compared to embryos electroporated with the control sgRNA (Figures 7a,b). We observed a similar loss of *GAD* > *Unc-76:GFP* expression upon targeting *Neurog* (Figures 7c,d), suggesting that *Neurog* is necessary for both pan-BTN and aBTN-specific gene expression. It was not clear if *Neurog* CRISPR completely abolished BTN fate or if it only affected BTN reporter expression. However, these data further support the conclusion that Neurog is required for BTN specification and effector gene expression during neurogenesis.

### Discussion of Predicted BTN Effector Gene Functions

Several genes upregulated by Neurog overexpression in the BTNs appear to be involved in neuronal function, especially neurotransmission, suggesting relatively early transcription of such genes relative to larval hatching. These include *GABA receptor (Gabrd)*, *Anoctamin 7 (Ano7), Neuronal calcium sensor (Ncs*), *Adrenergic receptor alpha 2 (Adra2*), *Synaptotagmin 7-related (Syt7-rel*), the neuropeptide-encoding *Ci-LF precursor (LF*; Kawada et al., 2011), and others. Even the canonical muscle myosin heavy chain gene *Myh-tun3* (previously known as *Ci-MHC3)*, a marker of adult body wall muscles in *Ciona* (Ogasawara et al., 2002), was unexpectedly found by *in situ* hybridization to be expressed in BTNs and other neural tissues. A neuron-specific function for the muscle myosin heavy chain gene *MyH7B* (which closely resembles *Ciona Myh-tun3* by sequence similarity) was identified in rats (Rubio et al., 2011), suggesting that perhaps a role for “muscle”-type myosins in neurons might predate the vertebrate-tunicate split. Due to our interest in understanding the delamination, migration, and dynamically polarized axon outgrowth of the BTNs, we focused our analysis on those genes hypothesized to be more directly involved in such cell behaviors, based on what we know about the functions of orthologs in other animals.

#### Cdk5 Regulatory Subunit (Cdk5r) and Doublecortin (Dcx)

**FIGURE 8:**
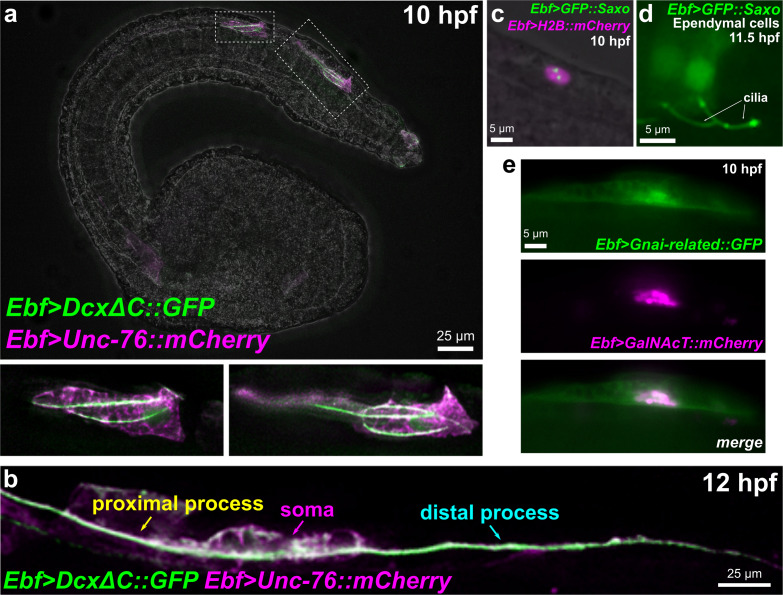
Subcellular localization of candidate effectors of BTN cell behaviors. **(a)** Embryo electroporated with *Ebf* > *Dcx*Δ*C:GFP* and *Ebf* > *Unc-76:mCherry* plasmids, showing GFP labeling of multiple microtubule bundles in the leading edge (the presumptive proximal process) of migrating BTNs. Insets magnified in bottom subpanels. **(b)** Embryo electroporated as in **(a)** but imaged at a later time point (12 hpf), showing a GFP-labeled microtubule bundle extending through proximal and distal processes (anterior is to the left). **(c)** GFP:Saxo (green) labeling putative centrioles/centrosome in a BTN cell. Nucleus labeled by H2B:mCherry (magenta). **(d)** GFP:Saxo is also seen in the cilia of ependymal cells of the neural tube/nerve cord. **(e)** Gαi-related:GFP (green) is enriched at the plasma membrane and around the Golgi apparatus, which is co-labeled with GalNAcT:mCherry (magenta).

Microtubule stabilization has been shown to be essential for neuronal migration and axon specification (Witte et al., 2008), though the mechanisms underlying its local control remain largely unknown (Kapitein and Hoogenraad, 2015). In vertebrates, Neurog1 and Neurog2 control neuronal migration in part through upregulation of *Cdk5r1* and *Doublecortin (Dcx)* effectors (Ge et al., 2006). Both *Ciona* orthologs of *Cdk5r1* and *Dcx* are upregulated in BTNs by Neurog, suggesting a conserved regulatory network for neuronal migration that is shared between *Ciona* and vertebrates. Cdk5r1 (also known as p35) is an activator of Cdk5, and the Cdk5/Cdk5r1 is required for microtubule stability in neuronal migration and axon outgrowth in several examples (Nikolic et al., 1996; Chae et al., 1997; Lambert de Rouvroit and Goffinet, 2001; Smith et al., 2001). Human DCX and the closely related Doublecortin-like kinases (DCLK1/2) are represented by a single ortholog in *Ciona, Dcx/Dclk* (referred from here on as simply *Dcx*). In mammals, Dcx has been proposed to be essential for neuronal migration and differentiation by nucleating, binding, and/or stabilizing microtubules (Corbo et al., 2002; Moores et al., 2004; Ettinger et al., 2016). The closely related vertebrate Doublecortin-like kinases are also associated with microtubules (Lin et al., 2000). While Dclk1 mutant mice show few neuronal migration defects, Dclk1/Dcx double mutants show extensive cortical layering and axonal defects, suggesting some overlapping roles for these paralogs (Deuel et al., 2006). Dcx/Dclk proteins contain two DCX protein domains, as does *Ciona* Dcx. As a proxy for the subcellular localization of this protein, we constructed a DcxΔC:GFP fusion comprised of the two DCX domains fused to GFP. When driven by the *Ebf* neuronal promoter (-2.6 kb upstream; Stolfi and Levine, 2011) in differentiating neurons, we observed DcxΔC:GFP enrichment in microtubule bundles extending into the leading edge of migrating BTNs at 10 h 20°C (Figure 8a). At 12 h 20°C, DcxΔC:GFP can be seen labeling a microtubule bundle spanning both proximal and distal processes (Figure 8b). This microtubule bundle localization suggests a conserved role for Dcx in *Ciona.*

#### Saxo: Stabilizer of Axonemal Microtubules

Positioning of the centrosome and associated Golgi apparatus has been shown to be an essential intrinsic cue for neuronal polarization (de Anda et al., 2010; Andersen and Halloran, 2012). However, this appears to be highly context-dependent and difficult to study *in vivo* due to the transient nature of centrosome position, tissue complexity in the developing CNS, and species- and cell-type-specific differences (Basto et al., 2006). Microtubule stabilization has been shown to be essential for axon specification (Witte et al., 2008), though the mechanisms underlying its local control remain largely unknown (Kapitein and Hoogenraad, 2015). Because centrosome repositioning is also driven by microtubule stabilization (Burute et al., 2017; Pitaval et al., 2017), this suggests that such centrosome-associated microtubule stabilizers might function as key effectors linking centrosome position and axon outgrowth. In the BTNs, initial axon outgrowth is concurrent with migration: the leading edge of the BTNs extends and becomes the proximal (anterior) process of the axon. Thus, polarization, migration, and axon outgrowth might be tightly coupled in the BTNs.

Previous MG neuron transcriptome profiling and a follow up ISH revealed that *Saxo (Stabilizer of axonemal microtubules)* was expressed in the BTNs, in addition to the ddNs (Gibboney et al., 2020). *Saxo* is the *Ciona* ortholog of human *SAXO1/SAXO2*, formerly *FAM154A/FAM154B.* These genes encode a highly conserved subfamily of STOP/MAP6-related proteins that stabilize microtubules (Dacheux et al., 2015). In human cell culture, SAXO1 localizes to centrioles and cilia and mediates stabilization of cold-resistant microtubules. They do so through 7 microtubule-binding/stabilizing “Mn” domains (Dacheux et al., 2015), which are conserved in *Ciona Saxo*. SAXO1/2 have not been implicated in neurodevelopment or cell polarity *in vivo*, but depletion of related MAP6 proteins in mice results in synaptic defects and schizophrenia-like symptoms (Volle et al., 2012).

A GFP:Saxo fusion when expressed in *Ciona* was found to localize to centrosomes in BTN precursors (Figure 8c), and to cilia of ependymal cells (Figure 8d), also consistent with a potentially conserved role in microtubule stabilization. Given its expression in both BTNs and ddNs, and given the dynamic repositioning of the Golgi apparatus observed in both these neurons types immediately predicting direction of axon outgrowth (Stolfi et al., 2015; Gibboney et al., 2020), Saxo is one of the more intriguing candidate effectors of neuronal polarization that remain to be functionally characterized.

How might extracellular cues impinge on centrosome position *in vivo?* One pathway that has been implicated in this process during neuronal migration is the Semaphorin/Plexin pathway (Renaud et al., 2008). We found that *Semaphorin 6 (Sema6)*, a class 6 Semaphorin orthologous to human SEMA6A/SEMA6B/SEMA6C (Yazdani and Terman, 2006) is expressed in migrating BTNs and broadly in other CNS neurons including those in the brain and MG. In mice, *Sema6a* and its receptor *Plexin A2* control migration in granule cells of the cerebellum, through regulating centrosome position and nucleokinesis (Renaud et al., 2008). In mammals, Sema6a can inhibit Plexin in *cis* as a mechanism to reduce sensitivity to Sema6a in *trans* (Haklai-Topper et al., 2010). Perhaps its expression in developing *Ciona* larval neurons reflects such a mechanism.

#### Gαi-Related

We identified a gene encoding a homolog of the small Gαi/o protein subunit family that by *in situ* hybridization was observed to be upregulated in migrating BTNs and notochord cells (Reeves et al., 2017). This rather divergent *G*α*i* gene (*KH.C2.872*, referred to simply as *Gnai-related*), is one of three *G*α*i/o* paralogs that seem to be *Ciona-* (or tunicate-) specific duplications: *KH.C1.612*, *KH.C2.872*, and *KH.L96.27.* Of these, *KH.C1.612* seems to be the original “founding” paralog, as it still retains exons/introns, while *KH.C2.872*, and *KH.L96.27* are both encoded by a single exon, suggesting possible duplication by retrotransposition, followed by subfunctionalization (Ohno, 2013).

In mammalian cells, upregulation of Gαi can act as a molecular “switch” to inhibit RhoA by competing with Gα12/13 proteins for interactions with the same G-protein coupled receptor (GPCR), resulting in the activation of Rac1 activation and increased cell motility (Sugimoto et al., 2003). This antagonism between Rho/Rac is also seen in delaminating neural crest cells, in which Rho inhibits Rac activity to keep cells in an epithelial state (Shoval and Kalcheim, 2012). In radial neuron migration, Gα12/13 proteins *terminate* migration (Moers et al., 2008), and have been shown to do so through RhoA in cultured neurospheres (Iguchi et al., 2008). Thus, transcriptional control over the relative expression levels of Gαi and Gα12/13 might be a common mechanism for regulation of neuronal migration, shifting between activation of Rac1 (promoting migration) or of RhoA (inhibiting migration).

Interestingly, we found that a Gαi-related:GFP fusion was enriched in or around the Golgi apparatus, in addition to the plasma membrane (Figure 8e). The localization of Gαi to the Golgi has been shown to be important for vesicle trafficking and the structural organization of the Golgi stacks (Lo et al., 2015). Furthermore, Golgi-resident Gαo regulates protrusive membrane activity (Solis et al., 2017). Given the dramatic reorientation of the Golgi apparatus during BTN migration and its relation to BTN neurite extension (Figures 1e,f), it will be interesting to further investigate the potential roles of *Gnai-related* in these processes.

## Conclusion

Here, we have used the BTNs of *Ciona* as a model in which to study the regulation of neurogenesis, both upstream and downstream of neuronal fate specification by the conserved proneural factor Neurogenin. More specifically, we have elucidated in more detail the mechanism by which FGF/MEK/ERK regulates BTN neurogenesis in *Ciona*, suggesting that a posteriorly localized source of Fgf8/17/18 spatially restricts sustained *Neurog* expression and subsequent specification of the aBTNs, but possibly not that of the pBTNs. This reveals close parallels with FGF-dependent regulation of neurogenesis in vertebrate spinal cord and neural crest, but also suggests a potential difference between very similar neuron subtypes originating from related but distinct cell lineages. It will be an interesting topic of future investigation to understand how the regulation of pBTN neurogenesis differs from both aBTNs and related neuron types in other chordates. Finally, we revealed the transcriptional dynamics of effector genes downstream of Neurog in the BTNs, identifying and validating several conserved genes that might be key for BTN delamination, migration, or polarization. Future studies will focus on the functions of these identified candidate effectors and the mechanisms by which they potentially regulate the dynamic yet invariant cell behaviors of the BTN precursors.

## Materials and Methods

### Embryo Handling and CRISPR/Cas9-Mediated Mutagenesis

Adult *Ciona robusta* (*intestinalis* Type A) were collected from San Diego, CA (M-REP). Dechorionated embryos were obtained and electroporated as previously established (Christiaen et al., 2009a, b). Constructs were made using previously published *Neurog −3010/−773* + *−600 [Neurog(BTN)]* driver to express *dnFGFR* (Davidson et al., 2006) and *CA-Mras* (Razy-Krajka et al., 2018), with an artificially inserted stop codon in front of the 3’ *Not*I restriction enzyme cloning site for some constructs where we wished to avoid fusion of N-terminal Neurog sequences with the transgene (e.g., dnFGFR). Cas9 and sgRNA expression vectors were constructed or used as previously described (Stolfi et al., 2014; Gandhi et al., 2017). Non-specific “Control” sgRNA sequence (target: CTTTGCTACGATCTACATT) used as previously published (Stolfi et al., 2014). *Fgf8/17/18* sgRNAs were validated as previously described, using loss of *Fgf8/17/18* > *Fgf8/17/18:GFP-*derived fluorescence as a non-quantitative screen for functional sgRNAs (Supplemental Figure 2). *Neurog* proximal promoter-targeting sgRNAs were validated by PCR amplification of the targeted region and Sanger sequencing as previously described (Supplemental Figure 3; Gandhi et al., 2018). Electroporations were performed as single biological replicates. Images were captured using Leica DMI8 or DMIL LED inverted epifluorescence compound microscopes. Plasmid and primer sequences not previously published and electroporation mix recipes can be found in the Supplemental Sequences File.

### FACS and RNAseq

Embryos were electroporated with the following combinations of plasmids: *70 μg Neurog −3010/−773 + −600 > tagRFP/tagBFP + 50 μg Neurog −3010/−773 + −600stop > Neurog (Neurog > Neurog condition). 70 μg Neurog −3010/−773 + −600 > tagRFP/tagBFP + 50 μg Neurog −3010/−773 + −600stop > Neurog:WRPW (Neurog > Neurog:WRPW condition), 70 μg Neurog −3010/−773 + −600 > tagRFP/tagBFP + 50 μg Neurog −3010/−773 + −600 > lacZ (Neurog > lacZ* “control” condition). Embryos were dissociated and FACS-isolated using a BD FACS Aria cell sorter into lysis buffer from the RNAqueous-Micro RNA extraction kit (ThermoFisher, Waltham, MA, United States) as previously established (Wang et al., 2018a, b). BFP + or RFP + cells were isolated with no counterselection. Cell numbers obtained were: Neurog > lacZ(control) replicate 1: 975 cells; Neurog > lacZ(control) replicate 2: 200 cells; Neurog > Neurog replicate 1: 284 cells; Neurog > Neurog replicate 2: 800 cells; Neurog > Neurog:WRPW replicate 1: 2418 cells; Neurog > Neurog:WRPW replicate 2: 114 cells. RNA was extracted from each sample according to the RNAqueous-Micro kit instructions. cDNA synthesis was performed as described (Wang et al., 2017), with SMART-Seq v4 Ultra Low Input RNA kit (Takara). Sequencing libraries were prepared as described (Wang et al., 2017), with Ovation Ultralow System V2 (NuGen). Libraries were pooled and sequenced by Illumina NextSeq 500 Mid output 150 Cycle v2, to generate 75 bp paired-end reads, resulting in 192,396,840 single-end reads for the 6 samples. Resulting FASTQ files were processed by STAR 2.5.2b and mapped to the *C. robusta* genome (Dehal et al., 2002; Satou et al., 2008). Output bam files were processed using Rsubread/featureCounts (Liao et al., 2013), with the parameter “ignoreDup = TRUE” to remove the read duplications resulting from library amplification. All reads after duplication removal that mapped to the exons of KyotoHoya (KH) gene models (Satou et al., 2008) were counted for differential expression analysis. Differential expression beween Neurog > Neurog and Neurog > lacZ, and between Neurog > Neurog:WRPW and Neurog > lacZ was measured by EdgeR (Robinson et al., 2010; Supplemental Table 1). Raw and processed sequencing data are archived at NCBI Gene Expression Omnibus (GEO) under accession ID GSE150913. All other processed data, scripts, and Supplementary Images can also be found at OSF at the project-specific link https://osf.io/uqfn2/.

### Embryo *in situ* Hybridizations and dpERK Immunohistochemistry

Adult *Ciona robusta* (*intestinalis* Type A) were collected from San Diego, CA (M-REP). Dechorionated embryos were obtained and electroporated as previously established (Christiaen et al., 2009a, b). Sequences of *in situ* hybridization probe templates can be found in Supplemental Table 3. *Neurog* perturbation and control plasmids were previously published (Stolfi et al., 2015). Probes were prepared either from published clones, synthetic DNA fragments (Twist Bioscience, San Francisco, CA, United States), or directly from RT-PCR amplicons (see Supplemental Table 3 for details). Probe synthesis and fluorescent, whole-mount *in situ* hybridization were carried out as previously described (Beh et al., 2007; Ikuta and Saiga, 2007). dpERK staining was carried out as previously described (Stolfi et al., 2011), using 1:500 mouse monoclonal anti-dpERK antibody (Sigma #M9692), and tyramide signal amplification.

## Data Availability Statement

The datasets presented in this study can be found in online repositories. The names of the repository/repositories and accession number(s) can be found in the article/ Supplementary Material.

## Author Contributions

KK, SG, WW, and AS designed the study and experiments. KK, SG, FR-K, and AS performed the experiments and collected and analyzed the data. EL and WW performed bioinformatic data analysis. AS supervised the study and secured funding. KK, SG, WW, and AS wrote and edited the manuscript. All authors contributed to the article and approved the submitted version.

## Conflict of Interest

The authors declare that the research was conducted in the absence of any commercial or financial relationships that could be construed as a potential conflict of interest.
