# Supplementary material for: Regulation of Neurogenesis by FGF Signaling and Neurogenin in the Invertebrate Chordate Ciona
Source: Front Cell Dev Biol. 2020 Jun 23;8:477. doi: 10.3389/fcell.2020.00477 (PMC7324659; doi:10.3389/fcell.2020.00477)
Supplement: Supplementary file 4 [file Data_Sheet_4.DOCX]

**Supplemental Sequences**

**Kim, Gibboney, et al.**

All driver>transgene combinations contained in the pCESAx plasmid (see below)

>Fgf8/17/18-2.1ksec_Fgf8/17/18::GFP

ggcgcgccgtctgctgcgaaggtttgtaaagtgaaacaattagtgggctactgcagttctactttctgtacaaatatacgctttatcacgctgtcaccttgacagacgttattgtaggaaagtttttgtgctgaacaatagccttgtaattagttgcgggggggcctctcggttcaaggccgaatagatacgacctctgatcagcgggtcgaaaacgaacgtaacaatagggaattatttcgtgctgtgacgtcggccgagtggggctgggaaccatttctaaaacattcctggcataatgatacgatgtaagcgcaatatttcacaaacgaaacaagtttacgtgactgtaactgcataagcgttgctttgtcactggaggcgccaaccgcacgataagatatttcagcgacggtcgcaatgaaaacggacaccctattacatgtatgacggccgttaatacctctgtcgctttgacaccttccaattgtcgataacataacccgcaacttgatattctgtgaatattatgacacccggcgccttttgcctgaattgtgtaatcgtgccgcaatattcgaaattgtcctgggtttatttttaaaggcagacgtcagagaaattgtaattctttacatcagatctgataaaccgctcgatttcacggatgtgttgaacaagcgccgtttcaaactgtcgcagttttatttttatcttcgtataaatttacagcggaattcattgaatgctaaactgttgtataaagaagtgtgcggaaagacgggccctgttgggacatactatcaaactattctgatcgtgttttaaacaattaacaacggtatatgggattcgcgagaaaacggtcttataatttttgaatattctttgtttactaccgaataggacaagaaaatagaatgaataggtgtcccatctttccccaccctatactatatatgaaccacacgtactccaaacacacttaacagcgtgacatatttcgctattgcgccagtttaaaacacttttatcttgagttcaataggcatacgtgtcatagttactacacacgttgtaacttttcccgtatcggaagttttttttaaaaattaggactctcctatgacgaattatattaattgctcccctggcgcggtttattttgtcttttcccacttattttcctttgcccacgttttatacagttacctttaatttgctacgcgtttgctcgacgggcggcatgagcgttttggggattatgacgtaacaaaacggtttttaatctcttcctctatgttacgacataatagcacctgtggaaaaacacaaacctgttttgttgggaagctgtatggttgtaaagggatgtgtgtttcgttttgttggtctctgctaaatacacgagtgcaatttcagtcaattacgaataccaacgtatgaaaagaatgcgcagattttgctaattggtttgtttaccctcgttcgtgcttggaattctgcaaattgttgttgtgtgggttagaacaatccaatcaccctgaaagtttttttaaatgcagaagaaaaccattaattttgtgcccaaaacaccgtaagctgtagccagcacaagttcgatatgtgttcatcaaaaatcggcggactttttctcaccgtcgcttttttcccgcggctccagcaacgcgtttgttaaaagcgcgttgcctaccgttatttacattaaacgatgccgatcgctgggcggttcgttatttttactcgtctggtaatggacaaaataacaacccagtcgtgtgcttagattttagcggtatggagcgccacgcgccgaccctcgggaaggacatgctgcacagcgggaaaccgagctttaggatccggcagataattttattcggagtcgatgttagaattgttataagttgttcaggataatagcaaagtgaaagcagaaataaatttaaactttatgctttgtatatttattcagtaagtagttaaataatgtcaattcggatttaatgcattgcgagtataaatagtaaatccgaatatcaaagtgatttctaagggacattattcttctcttggattacatacgaaaatacgaccctccgcggccgcAACCATGCGACCCTCCATTTGCAAAGATATCTACGCGAACCTGTACAGGTTCTTGCTGGGTATCATCGTTCTCGGAGACATAGCCAGCGGGATTCCGCCAAATTACATCAGAGATTACATTAAAAGCGAAATATTTCAAGAGAACGTAAGCCAATCTTCTTCCACGAGAAGATACTGTGAGATTTACTCGAAACGAACCTCGAACCACATTCGAATACTCGGCAAGAAAGTGGACGGGAGAGGAAGAAGAGGCAGCAATCATGCAAAACTAATAATCCACTCTAAAGGCTTATGGGGTCGGATAATGATTCAAGGCGTCCAAACTGAACGGTTTCTTTGCATGAATAAAAGAGGACGACTgGTTGGAAAGAATTATCGAAAAGCGATCAACGACCCGAAGTGCCTATTCTTCGAAGAGATGTTGGAAAATTATTTTGATGCTTTTTCGAACTCTAAGTTTCGCGATTGGCATATTGGTTTCCATTCAAACGGCAGACCAAAGCGAGGTCCACACACCAAAGAAATCGGCACTGATGAAATGTTCATAAAGAAGTGTACAGCATCCAGCGGTATGGTACCGAACCCGACCGCTGCCAGTAACGAGAAGTACAAATGGATTATTGGCGAGTTCGAAAGGGACGTGAAGCGACATAACAAGAAGAAAAAGAGAGCCCCTTTATCTGTTTTAAGACCAAATTATTCTGTCACTAGAGATTCATCAACCGCCGACGAAGTTATGCTTGCCGACTCTTCCAGAACTTTGTCGACCCTACCTCCGTATGTTCAAGCTGAAATCGAACGACGACGGAGTCTGTACCCTGTTTTGCGACAGCAGCGTAGACTTGACGTTACCATACCGCCAGCAGGTGGGACAGTGTCCGCCATGGATCCGCTGTACTCGAGCCCAAAGCATCGAAAGTTACCTCGCCGCAATAAACCTAGACGGAGCAGACAAAATAAGAAACGAAAACACAGAAGACATAAATCGAAATGGAGGTCTACTGTCCCTACTGATATAGTGGATTCAGACTTTCAACCTACGACTGGGACCTTTACAACACCGTTCCCAATAAACTCTGTAAGCGAACCCGAAGCCGGGTTTATTAGAACCCCGACTACAGCACCGGGCCTGCATAGACAACGATCGCGGAGCGGGAGGCGGCGACCGAAACAGAGAAGACGGACGAAAAAAGTCCATGGTCGGAAACAGAAAACCGGTCGGCGGAACCGAAAACGAGCAAACACAactagtaccatggtgagcaagggcgaggagctgttcaccggggtggtgcccatcctggtcgagctggacggcgacgtaaacggccacaagttcagcgtgtccggcgagggcgagggcgatgccacctacggcaagctgaccctgaagttcatctgcaccaccggcaagctgcccgtgccctggcccaccctcgtgaccaccctgacctacggcgtgcagtgcttcagccgctaccccgaccacatgaagcagcacgacttcttcaagtccgccatgcccgaaggctacgtccaggagcgcaccatcttcttcaaggacgacggcaactacaagacccgcgccgaggtgaagttcgagggcgacaccctggtgaaccgcatcgagctgaagggcatcgacttcaaggaggacggcaacatcctggggcacaagctggagtacaactacaacagccacaacgtctatatcatggccgacaagcagaagaacggcatcaaggtgaacttcaagatccgccacaacatcgaggacggcagcgtgcagctcgccgaccactaccagcagaacacccccatcggcgacggccccgtgctgctgcccgacaaccactacctgagcacccagtccgccctgagcaaagaccccaacgagaagcgcgatcacatggtcctgctggagttcgtgaccgccgccgggatcactctcggcatggacgagctgagtaagaattc

Fgf8/17/18-2.1ksec driver Fgf8/17/18 coding sequence GFP

>Ebf-2.6kSTOP>DcxDeltaC::GFP

ggcgcgccattcttccgggaaataagagcggcagcgactttattcgaatttcaaatttccatgatattcgataccccacactccaccctataacggtgacctggtttaggaaatatgtgacgttgcttcacgacaattttagcagctatataagttaccattgcaaaaaagatcagtctttttatttatcacaatttttaccagatttttaaatattttttagaaataacttaaattttgatcaaactggattaaaattgcaaattttacatttttgtgataaatattaatctttttagtcgccatggcttaaaaatgtcatttgtttagtccgctgtattcaattttaccgtttgataaatcaaacggttaacttgcgcataaaaccaatttctcatatggatattactccgcgcgagttctaacaacgcgttctcttcgctggccaagatcgatactccgtgacgtcacaatcacatgcgcgcgtctgaaatatggtcgacagagttgcaacacgcaacccttttgtttgggttaatttccctctttgttttttaagtccgatcgcgtcgccacatctgttcgacgaatcttcgttctgtcttagcaactttgcggctctgtccattgtgaagtcgtaaacgaggcattgtcgtcactgctgtctcgcctacgtcacaaagcagtgcacagtgacgtcacagagacgaggtcgcgtgccttcgagttggaaaattcaaagcattgcactttttgccgaatgttaatttttacgacggaagaagttaggacaagcataaaataacaaataaataacccataaaaaacgtaaaaacaaacaagagaacggtattcaaaattagaatttcgaaaaaaatgttaaaaaataatacttagagtcgctgaacaatttaagccgacaaaggccacaaaactgcctaaaatttataaaaaaaatgtaaaattattttgttttttttaaactacagttatcacctttaaaacaaacaaattagcaaacgttgtaattacttcacaactttcttgcgacgctaaaaggcggcgaattttattgctattgtgacgtcacaagcgctctcgtcacgcccggatacgattagaacaacgaaggattgtttgtttttaattatttctctgtttaatcatttgatttagcgcggcacaaattttgttttatataaaatgtatccattttatctctgcgcgtttttgtactattttttgaaaaatgtttgttaaccttttgaaaatcgcgaaaccaacgaaattatttccaaaggctgtacaattctttttcgttaggttacgtgtttaagtataggcccaagttttaaatggcggacagagtttcgtattttgatatttgaatttttttgaaatcttgaaaaaaaataattgtacgttttacatagaataactaaccaaaatatctgaagcaaaaattacgtacaattttttaaaatgtaatttactttctagcttttaatttttgtgcttttctcaatatgtgtgccatattttaaaaacgtaaatttgcctgttgttaagcggaggaaaaagtaatctgcgtgaatgcgaaaaacacgattttggaatcagcgccggcaatggtgtttgtaaataggggtggcatacgcgtttcgtagcgaaagagagaatgaggcgaaagtcgacagatgcacgctccgatttatgagacaggaaccagtcgcgagggcacggaggaaaaagaaccttactccaagacatgcgccgccttttttctttctgtcctagtcaggaatactagagtatagaaggccacgcgtcgtggagtttaaaaccagcaagccagtgtctcaacggacacatcaatacagagacttctctcagtggacaactcggacgattcgccactaacttggtggattcgtcccggacgacccatgggccgggtcccagcgcgctagttggccaccatacagtgtagaatcagctagatcgtctctgcggatttcgcaaatagattgagttggagatagcttcccgaccgggatttcgactaatttgcaatgttagttattaatcaaggtgacagtcaggagttaaagttaatttaccttttgaaagggcaaaaagattttcgaagtaaattgattccgttaattgtaactcttaaacgcaaaccgataaacgacgccattttgcttttcattgagaaactaaccatttaggcattctataattaaaattaaatgtttttaaaatctgtaactatctgtaataaactagaattatttatttcagtttaaatttttatttaaaacaataattttattatttcctttattcatcctaatgatattggtatagagaaaacgatgtttttatttccataaaattttatttcagaaaagtcattttgttcaatttaaaaaattcctttattctaaaaaaatgcccaaaacaagttctttatttcttaaaaattatcctaaataaaaaaatccacgttttaataaaatgtataaaattgaaaactataaaaagatgacttatttttttaccctaacgtgatttttcaccagataccttaagtgttattttatttgtaagtaatatccaaatggcaacaatcgcgtaagcggccgcaaccATGGCAGAACAATTTGGTGGGGAATCAAAAGGTCGAAGAAAAAGTAGTGGCTTGTCCAGAAACCCTAGTTATGGCTCGCAGAACAATTTGTACAGGGCCAGATCCTTACAAAGAATCGCCGACGAAAAAAGAGCAAAAAAGGTTCGGTTTTATCGAAATGGTGATAATTTTCACACCGGACTTGTGTACGCTGTTTCCACCACACGTTTCCGTACATTTGAAGCAATGCTTATTGATCTTACCAACCGTCTTGCTGATAAAGTACATCTGCCTCATGGTGTTAGGGTTGTGTTCTCTTTAGATGGGGCGCGGAAGATTGAAACTATACAAGAACTTGAACATGGTGAAAGCTATGTATGTGCTTCATCAGACATGTTTAAACGTCTTGAATACGGACGTAACACCAACCCTAGCTGGTGTGTAAATGTTCGAACGGGTAGTTCTGCGAGCTGTGTGAGCCACGAGTCGCTCTGCAGGAATCTTCCCCTAGAGCGGGAGAGCCGGGACTTCATCAAACCAAAGCTCGTCACCATTATAAGGAGTGGAGTAAAGCCCCGAAAAGCGGTTCGAATaCTGCTGAACAAGAAAACTGCTCATTCTTTGGAACAAGTTTTAAATGATGTAACAAAAGCAATTAAGCTTGACACTGGTGCTGTGAGGAAGGTGTATACTCTGCAAGGCAAGCAGGTTCAAAGTCTGCAAGATTTCTTCGGTAACGACGATATATTCATCGCATATGGACACGAGAAACTTTCCCAAGATGATTTTGACATTGATGCAGATGAAGCGAAATTCATTCGCCCTTATTCATCACAAACTGCCCGTACGCCTCGTGTTACATTACAAAGAAAAGGCAGCGCCCGTCTCAACCGAACCGCGGATTTTCCAACATCGCCACAAGGCAAGGCCGCACCTCGTCGGGCACAATCACTACGGATCTCACCCACGCATCATAAGAAACCGCTGTCCTTGCATAGCGGCAGTATGCTGGAAATGGACACGTCGCCTTACCCGCAGCAAATCCATGAACGTgctagcaccatggtgagcaagggcgaggagctgttcaccggggtggtgcccatcctggtcgagctggacggcgacgtaaacggccacaagttcagcgtgtccggcgagggcgagggcgatgccacctacggcaagctgaccctgaagttcatctgcaccaccggcaagctgcccgtgccctggcccaccctcgtgaccaccctgacctacggcgtgcagtgcttcagccgctaccccgaccacatgaagcagcacgacttcttcaagtccgccatgcccgaaggctacgtccaggagcgcaccatcttcttcaaggacgacggcaactacaagacccgcgccgaggtgaagttcgagggcgacaccctggtgaaccgcatcgagctgaagggcatcgacttcaaggaggacggcaacatcctggggcacaagctggagtacaactacaacagccacaacgtctatatcatggccgacaagcagaagaacggcatcaaggtgaacttcaagatccgccacaacatcgaggacggcagcgtgcagctcgccgaccactaccagcagaacacccccatcggcgacggccccgtgctgctgcccgacaaccactacctgagcacccagtccgccctgagcaaagaccccaacgagaagcgcgatcacatggtcctgctggagttcgtgaccgccgccgggatcactctcggcatggacgagctgagtaagaattc

Ebf -2.6kb STOP driver Dcx Delta C-terminus GFP

>Ebf-2.6kSTOP>GFP::Saxo

ggcgcgccattcttccgggaaataagagcggcagcgactttattcgaatttcaaatttccatgatattcgataccccacactccaccctataacggtgacctggtttaggaaatatgtgacgttgcttcacgacaattttagcagctatataagttaccattgcaaaaaagatcagtctttttatttatcacaatttttaccagatttttaaatattttttagaaataacttaaattttgatcaaactggattaaaattgcaaattttacatttttgtgataaatattaatctttttagtcgccatggcttaaaaatgtcatttgtttagtccgctgtattcaattttaccgtttgataaatcaaacggttaacttgcgcataaaaccaatttctcatatggatattactccgcgcgagttctaacaacgcgttctcttcgctggccaagatcgatactccgtgacgtcacaatcacatgcgcgcgtctgaaatatggtcgacagagttgcaacacgcaacccttttgtttgggttaatttccctctttgttttttaagtccgatcgcgtcgccacatctgttcgacgaatcttcgttctgtcttagcaactttgcggctctgtccattgtgaagtcgtaaacgaggcattgtcgtcactgctgtctcgcctacgtcacaaagcagtgcacagtgacgtcacagagacgaggtcgcgtgccttcgagttggaaaattcaaagcattgcactttttgccgaatgttaatttttacgacggaagaagttaggacaagcataaaataacaaataaataacccataaaaaacgtaaaaacaaacaagagaacggtattcaaaattagaatttcgaaaaaaatgttaaaaaataatacttagagtcgctgaacaatttaagccgacaaaggccacaaaactgcctaaaatttataaaaaaaatgtaaaattattttgttttttttaaactacagttatcacctttaaaacaaacaaattagcaaacgttgtaattacttcacaactttcttgcgacgctaaaaggcggcgaattttattgctattgtgacgtcacaagcgctctcgtcacgcccggatacgattagaacaacgaaggattgtttgtttttaattatttctctgtttaatcatttgatttagcgcggcacaaattttgttttatataaaatgtatccattttatctctgcgcgtttttgtactattttttgaaaaatgtttgttaaccttttgaaaatcgcgaaaccaacgaaattatttccaaaggctgtacaattctttttcgttaggttacgtgtttaagtataggcccaagttttaaatggcggacagagtttcgtattttgatatttgaatttttttgaaatcttgaaaaaaaataattgtacgttttacatagaataactaaccaaaatatctgaagcaaaaattacgtacaattttttaaaatgtaatttactttctagcttttaatttttgtgcttttctcaatatgtgtgccatattttaaaaacgtaaatttgcctgttgttaagcggaggaaaaagtaatctgcgtgaatgcgaaaaacacgattttggaatcagcgccggcaatggtgtttgtaaataggggtggcatacgcgtttcgtagcgaaagagagaatgaggcgaaagtcgacagatgcacgctccgatttatgagacaggaaccagtcgcgagggcacggaggaaaaagaaccttactccaagacatgcgccgccttttttctttctgtcctagtcaggaatactagagtatagaaggccacgcgtcgtggagtttaaaaccagcaagccagtgtctcaacggacacatcaatacagagacttctctcagtggacaactcggacgattcgccactaacttggtggattcgtcccggacgacccatgggccgggtcccagcgcgctagttggccaccatacagtgtagaatcagctagatcgtctctgcggatttcgcaaatagattgagttggagatagcttcccgaccgggatttcgactaatttgcaatgttagttattaatcaaggtgacagtcaggagttaaagttaatttaccttttgaaagggcaaaaagattttcgaagtaaattgattccgttaattgtaactcttaaacgcaaaccgataaacgacgccattttgcttttcattgagaaactaaccatttaggcattctataattaaaattaaatgtttttaaaatctgtaactatctgtaataaactagaattatttatttcagtttaaatttttatttaaaacaataattttattatttcctttattcatcctaatgatattggtatagagaaaacgatgtttttatttccataaaattttatttcagaaaagtcattttgttcaatttaaaaaattcctttattctaaaaaaatgcccaaaacaagttctttatttcttaaaaattatcctaaataaaaaaatccacgttttaataaaatgtataaaattgaaaactataaaaagatgacttatttttttaccctaacgtgatttttcaccagataccttaagtgttattttatttgtaagtaatatccaaatggcaacaatcgcgtaagcggccgcaaccatggtgagcaagggcgaggagctgttcaccggggtggtgcccatcctggtcgagctggacggcgacgtaaacggccacaagttcagcgtgtccggcgagggcgagggcgatgccacctacggcaagctgaccctgaagttcatctgcaccaccggcaagctgcccgtgccctggcccaccctcgtgaccaccctgacctacggcgtgcagtgcttcagccgctaccccgaccacatgaagcagcacgacttcttcaagtccgccatgcccgaaggctacgtccaggagcgcaccatcttcttcaaggacgacggcaactacaagacccgcgccgaggtgaagttcgagggcgacaccctggtgaaccgcatcgagctgaagggcatcgacttcaaggaggacggcaacatcctggggcacaagctggagtacaactacaacagccacaacgtctatatcatggccgacaagcagaagaacggcatcaaggtgaacttcaagatccgccacaacatcgaggacggcagcgtgcagctcgccgaccactaccagcagaacacccccatcggcgacggccccgtgctgctgcccgacaaccactacctgagcacccagtccgccctgagcaaagaccccaacgagaagcgcgatcacatggtcctgctggagttcgtgaccgccgccgggatcactctcggcatggacgagctgtacaagactagtAAGTCTCAAAAATGGTGGCACAGACCTCGGCCaGCAGGTTTTCTCAACTTTGGCCACCAAGCGGGCAGCGGTACAGAATATGTTGACGAATATGTTCGACATAGAGTCCCTCCTACAGAATCGTTTAAACCAGCTGAAGAAATGCGAAAATCGGACGCAAAGGTGTCAGACGAGACAACTTTTAGACTTGATTACATTCCACACCAACTTTCAAAGCATGAGCCACACCCAAAAGAAGTGTACAACCCACCAGGCACGCCTATGGAGGGTGTAAGCATGTACAGACAAGACTACCCGGGTCATAACACAGGTCCTGCTCAaCTAGCGAAACGGTCGGAGGCAAGAAGTGTGCCTCTGGTAAAATTCGAAGCTCATCCTACTTATGCgAGCGACTTTAAACGTTGGACAATACCTCCGCGTGTGAAGTTGGGTCCCGACAACGATTACAAAAAGCCGGTTGTAAAAATGGAGAATACTTCCACTTTTCAACAGGACTACATTCACCGCTTCGCGCCACCACGAGAATCAGCGAGGCCGCCAGATAAAGCATTCCAGTCCGACGTCCCGCTGGAAAGCCATACAGTTCACCGGGTGAGCTTCATACCGCATCAGATACAACCAAGGTTGCAGCGGGAAAAAGAGAAGTACTGCGCGCCTACCGTTCCCATGAACTCCGAGACCACGTTCAAGCAAGATTTCACCGGACCAAGAGCCCTCCCGGCAGAGAGCATGAGGCCATCGCAAGCACCTTTCGTTTCAACAGATCCATTGGCGTCTTCGTCTGAgTTCAGAGACAGTTTTGTCGCCTGGCCCGTCGTGCGTCCCTATCGTAAAGAGCCTTTGAAGTATCAGGGCCCACAAGGCGATATGGAGCTTCTCTCCACACAAAGATTGGATTTCAGGTCTCTGAATGGCCGTCCCGCATCTGCAAAGCGCCCGGCCGTAAGAAGAGGTAAAGTCATGCCCTTCGAAGGAGTCACAAACTACAGCAGTGACTTTAAGAAGTGGAACGTCCCAAGAACGCTTGGTAAACCTCGTCCAGAGGCCATCCAGCAAACTGGGAGATTTGAAGGGCTATCCACCGCCCGTCAGCACTTCATCACACACAGCGGTGCTCTACCTGCCAGATCATGCAAACCAGACAACCGAGCTTTCCTCAGTGATTCTGCCCTCGAAGATAAAACGATTTACAAAGTGAGTTACGTCCCGAAATCCATGCGCGAAGTCGAGCGCTACCCAACACCGGATTGGCTGAAACAACAAGAAGATATTTGGAGGAAGACTGGCATGATGACACAAAAGACCAGAGGGATGACCCCCGAAGCTACCGCTGCGTATATTCGAGCTGCAGCATAAgaattc

Ebf -2.6kb STOP driver GFP Saxo coding sequence

>Ebf-2.6kSTOP>Gnai-related::GFP

ggcgcgccattcttccgggaaataagagcggcagcgactttattcgaatttcaaatttccatgatattcgataccccacactccaccctataacggtgacctggtttaggaaatatgtgacgttgcttcacgacaattttagcagctatataagttaccattgcaaaaaagatcagtctttttatttatcacaatttttaccagatttttaaatattttttagaaataacttaaattttgatcaaactggattaaaattgcaaattttacatttttgtgataaatattaatctttttagtcgccatggcttaaaaatgtcatttgtttagtccgctgtattcaattttaccgtttgataaatcaaacggttaacttgcgcataaaaccaatttctcatatggatattactccgcgcgagttctaacaacgcgttctcttcgctggccaagatcgatactccgtgacgtcacaatcacatgcgcgcgtctgaaatatggtcgacagagttgcaacacgcaacccttttgtttgggttaatttccctctttgttttttaagtccgatcgcgtcgccacatctgttcgacgaatcttcgttctgtcttagcaactttgcggctctgtccattgtgaagtcgtaaacgaggcattgtcgtcactgctgtctcgcctacgtcacaaagcagtgcacagtgacgtcacagagacgaggtcgcgtgccttcgagttggaaaattcaaagcattgcactttttgccgaatgttaatttttacgacggaagaagttaggacaagcataaaataacaaataaataacccataaaaaacgtaaaaacaaacaagagaacggtattcaaaattagaatttcgaaaaaaatgttaaaaaataatacttagagtcgctgaacaatttaagccgacaaaggccacaaaactgcctaaaatttataaaaaaaatgtaaaattattttgttttttttaaactacagttatcacctttaaaacaaacaaattagcaaacgttgtaattacttcacaactttcttgcgacgctaaaaggcggcgaattttattgctattgtgacgtcacaagcgctctcgtcacgcccggatacgattagaacaacgaaggattgtttgtttttaattatttctctgtttaatcatttgatttagcgcggcacaaattttgttttatataaaatgtatccattttatctctgcgcgtttttgtactattttttgaaaaatgtttgttaaccttttgaaaatcgcgaaaccaacgaaattatttccaaaggctgtacaattctttttcgttaggttacgtgtttaagtataggcccaagttttaaatggcggacagagtttcgtattttgatatttgaatttttttgaaatcttgaaaaaaaataattgtacgttttacatagaataactaaccaaaatatctgaagcaaaaattacgtacaattttttaaaatgtaatttactttctagcttttaatttttgtgcttttctcaatatgtgtgccatattttaaaaacgtaaatttgcctgttgttaagcggaggaaaaagtaatctgcgtgaatgcgaaaaacacgattttggaatcagcgccggcaatggtgtttgtaaataggggtggcatacgcgtttcgtagcgaaagagagaatgaggcgaaagtcgacagatgcacgctccgatttatgagacaggaaccagtcgcgagggcacggaggaaaaagaaccttactccaagacatgcgccgccttttttctttctgtcctagtcaggaatactagagtatagaaggccacgcgtcgtggagtttaaaaccagcaagccagtgtctcaacggacacatcaatacagagacttctctcagtggacaactcggacgattcgccactaacttggtggattcgtcccggacgacccatgggccgggtcccagcgcgctagttggccaccatacagtgtagaatcagctagatcgtctctgcggatttcgcaaatagattgagttggagatagcttcccgaccgggatttcgactaatttgcaatgttagttattaatcaaggtgacagtcaggagttaaagttaatttaccttttgaaagggcaaaaagattttcgaagtaaattgattccgttaattgtaactcttaaacgcaaaccgataaacgacgccattttgcttttcattgagaaactaaccatttaggcattctataattaaaattaaatgtttttaaaatctgtaactatctgtaataaactagaattatttatttcagtttaaatttttatttaaaacaataattttattatttcctttattcatcctaatgatattggtatagagaaaacgatgtttttatttccataaaattttatttcagaaaagtcattttgttcaatttaaaaaattcctttattctaaaaaaatgcccaaaacaagttctttatttcttaaaaattatcctaaataaaaaaatccacgttttaataaaatgtataaaattgaaaactataaaaagatgacttatttttttaccctaacgtgatttttcaccagataccttaagtgttattttatttgtaagtaatatccaaatggcaacaatcgcgtaagcggccgcaaccATGGGATGCGCCTCTTCTACATCGGGAGATGCGATGGCAGCCACCAGGTCCCGTCAGATAGACAGGGGCATCAAAGAGGACGCGAAACACGTCGATAAAGAAGTGAAGCTTTTGCTTCTGGGAGCTGGAGAGTCAGGGAAAAGTACAATCGCAAAACAAATGAAAATCTTGTACAAAGATGGCTTCTCCGAAAAAGAACGAAACTCTTACAGGCATGTGGTTTACGCCAACGTTGTACAGTCGATCGTTGCCATCGTAAAAGCGATGCCCACTCTTCAGATCGAATTTGATGATCCAAACTGTACTGAAGACGCAAAAAAGGTGCTCACTGCTGGGCAGATGTTGGGAGACTTTGAGCTCTCCGGGGAGATTGGAGATGCAGTAGCGAGGCTCTGGGAAGATCCAGGTCTCAGAAAGAGCTATGAGCGTTCCCGGGAGTACCAGCTTAATGACTCGGCCGAATATTACCTCGAATCGATAGAACGATTAAGTCAAGCCGACTATATGCCAACCGAGCAGGATGTCCTACGAACCCGAGTTAAAACTACTGGTATCACAGAGACGAACTTTAATTATAAGAACCTCGAGTTTCGGTTGATTGATGTCGGAGGACAGAGATCAGAGAGGAAGAAATGGATTCACTGTTTTGAGGATGTGACAGCCATTATATTCTGCGTCGCTCTTTCCGCTTACGATCTTCTACTTGCGGAGGACGAAGAGACAAATCGAATGCACGAGAGCATGAGGTTGTTCGACTCCATCTGCAACAACGCTTGGTTCCGAAGTACGTCGATGATTCTATTCCTGAACAAAACCGACATCTTCAGGAGTAAGATTAAACACTCTCCACTTAACGTGTGCTTCCCGGACTATCAGGGAGAAAACACATTCGATCAAGCTGCCACCTACATCCAGCTTCAGTTCGAAAACCTGAACAATCACCCAGAGAAGATGGTTTACACTCACTTTACATGCGCCACTGACACTACCAACGTACAGTTCGTATTTGACGCGGTTTCTGACGTCATTTTGACAAAGGTGCTTGGAGACTTGGGGCTTATGACAGAGactagcaccatggtgagcaagggcgaggagctgttcaccggggtggtgcccatcctggtcgagctggacggcgacgtaaacggccacaagttcagcgtgtccggcgagggcgagggcgatgccacctacggcaagctgaccctgaagttcatctgcaccaccggcaagctgcccgtgccctggcccaccctcgtgaccaccctgacctacggcgtgcagtgcttcagccgctaccccgaccacatgaagcagcacgacttcttcaagtccgccatgcccgaaggctacgtccaggagcgcaccatcttcttcaaggacgacggcaactacaagacccgcgccgaggtgaagttcgagggcgacaccctggtgaaccgcatcgagctgaagggcatcgacttcaaggaggacggcaacatcctggggcacaagctggagtacaactacaacagccacaacgtctatatcatggccgacaagcagaagaacggcatcaaggtgaacttcaagatccgccacaacatcgaggacggcagcgtgcagctcgccgaccactaccagcagaacacccccatcggcgacggccccgtgctgctgcccgacaaccactacctgagcacccagtccgccctgagcaaagaccccaacgagaagcgcgatcacatggtcctgctggagttcgtgaccgccgccgggatcactctcggcatggacgagctgagtaagaattc

Ebf -2.6kb STOP driver Gnai-related coding sequence GFP

>Neurog-3010/-773+-600**STOP** driver

ggcgcgccgtctgtttccgcatacatgcaatatgcacgcgttcattataatatcaagttttaccaatttccgtaaatttttaatggaacagtgtaattaacccgactatattaccgttttccgatcgcattgttacgtcacaataaaaccgcggtgtgttctgcgaagttaggaagtatctcattacgtcataatcgtcggatagttttgattgttttatcataatgcggactaagtagtccattactacaccgacgtaatcgccactttgtgacgtcacaataccgcaccagatcaatgacgtcatcaaaggggaaactcggaagtaataagtgacgaaatcaaaggcggaattatgacgttttcgcttttgattgttacgtacgtgggaaagagcaaacgaatcgggaaacgttttgctatcgtagattgtgacgaaataatgaaatagattgtgacgtcatgatgatttttatctccgaataaacggaatcgagttatcagcacacgcgtcacgaacattgtttaccaactgaatacaattgttaagttatttaaataaattttaatcaccttaaaccgagctgaattacacgccgatcaaacatatattatttgttttactttgtcgttacatttgtacttgctaaagcaaattcgctttaaaaaacggcccttctgaaagctttgactggtaaatgacatcaaagctgcggcgtttaaaagctgtttactgttcgttgtttaatcggtacagtgtttggtgtttattctatactccagtctggtttattttatttattttacaccgttttggttaattttgttgcgtaatttaaaatctggtacactgtttgtcccgtatttccctacccgcattacgagacctgattttacgctgtgtgacgtcacgccatatggcacaaaatgcaactttaaacgaataagcagcgtccacaaaatacatatcgggtgtattgcgtcgcgctaattgctcagatttccggaagtataatatttcaaccaatctctgcttattaaaacgttagattgactaattagaaaccacgaataatgttaattgctccaacccagtggttcattaattatgggttgtctaaaataaatgttggccaacacatttcagaaaattaagaattccaaagtgttaatcacccacaaagtaacatacgtagtagctcgtaagcgggcacgaggtgtatgaaacagaacacccgtgttataacaactgccatcatagatatctaaacatatgtttagatatctatgctgccattactccgcaatgcgcggataaataaattacattcattcgtatttgcaaaaatggtgtttttatgtctgtacggtccgggcataaagacgacagattgccacgcatgcagtcgggatcaattatttataaataatatgaattattcacgaggaatggcgctttcctgagccccgtgatttaggaaaatttgagaaaaatcacaaaaataaattacgaagtcgaaaatggagaaattttgggtaatttttatcatgaaatatgacatgcgtccaaattcgtgtatagtaggttggggtaaagtggtccatgtttttattctatttgtcgtctcatttagtagtaaacaaagaatattcacaaaattataactgtatccttgcgactctaaacaagcgttgttggttgttacaaaacgacctggaattatgggatattttgtgctgtacccatcttaccccacgatgctataaattctgattaaaaagcgacaagtttgtcaatagtaatttggagcttataattgacgaaacaaagtaatggttcggtttataaatgaaaataattgaaatatatactggaacattctttatcgattaggagaatgacttttgaaaaatataaataatttctttataaaatcgggttggaaaatagaaataaaataagacagtaatgtgccggtatacattttttctatattgattaaaattgaaacgtaaaataaaattgtcaaaacgcaaagtggaatacagcgtaagacaataaatgtaattggacagttacggcttagtttaattgccataattttgtttactaccaaatggaacggtaaagtagaataaagtcctggaccatcttacccaacctatactgtatttaaagacgacgtaaattggcatgtagtcgcatttaaattctgagcaatattttgtgtctctcggcagcgcgcatgaggttcggcgtataaggctagcggtcatgctttgttacgtcccagagtcaaggtcaggtattggcccggtcgtctgttcggtttttgagattcgtttccactcggaaaacgctattgtttcatcgctatccatttgtttgctaacgtctaagtcgccgcccgcatttgctggaagatttgccttttttctccgatttgtgacgtcacagtgcttcggctgatctctctgtttcatcccgatgcgcctttttctctactttctctcggagctatataagcagatcgtaatttctactttcactataatctgttttggttctacagagcccagtggctcatatttgcagaatgcattcgcagcatttctggcaaatatttcgtattaacaaaacctgtcaggccagggctttttgcaaaaggcattgtgacatatattttgtatatgtcaaactctagctaaagtttaaagttattcaaaactattttaggaaagtacggagagcagaataccatattttattgctaaaatttctttttcttcagaaacttaacaaatatcttatttcatttcagatttcaatcttgatattataaaaattttgctcttgctacaaaatgttggatTAAgcggccgc

Neurog -3010/-773 + -600 (“Neurog[BTN]”) Artificially inserted STOP codon

Fgf8/17/18 sgRNA (N)19 target sequences:

FGF8.ex5.439: AGCTGAAATCGAACGACGA

FGF8.ex5.201: AGAAGTGTACAGCATCCAG

FGF8.ex5.138: CAAACGGCAGACCAAAGCG

Neurog sgRNA N(19) target sequences:

Neurog.1: CTTACCATTACGTCTTGTG (Gandhi et al. 2017)

Neurog.p1: TGTCTGGCGATAGTATACG

Neurog.p2: ACGTAACAAAGCATGACCG

Primers used for PCR and Sanger sequencing for mutagenesis efficacy of Neurog.p1 and Neurog.p2 by “peakshift” assay (Gandhi et al. 2018):

Forward: AAAGTCCTGGACCATCTTAC

Reverse: CTTCTAGTGCGTCATTAAGACC

>Neurogenin gene and targets

tgtgccggtatacattttttctatattgattaaaattgaaacgtaaaataaaattgtcaaaacgcaaagtggaatacagcgtaagacaataaatgtaattggacagttacggcttagtttaattgccataattttgtttactaccaaatggaacggtaaagtagaataaagtcctggaccatcttacccaacctatactgtatttaaagacgacgtaaattggcatgtagtcgcatttaaattctgagcaatattttgtgtctctcggcagcgcgcatgaggttcggcgtataagagaaccgaatgcactttcccacagaagaagcggtgaatataatacagacatagtaatgtctggtgccgcagcaaaattcccaggttcccatgcatatgtcccCGTATACTATCGCCAGACAaggtaactttcccactcaaaaaaacggtgtccaaccgCGGTCATGCTTTGTTACGTcccagagtcaaggtcaggtattggcccggtcgtctgttcggtttttgagattcgtttccactcggaaaacgctattgtttcatcgctatccatttgtttgctaacgtctaagtcgccgcccgcatttgctggaagatttgccttttttctccgatttgtgacgtcacagtgcttcggctgatctctctgtttcatcccgatgcgcctttttctctactttctctcggagctatataagcagatcgtaatttctactttcACTATAATCTGTTTTGGTTCTACAGAGCCCAGTGGCTCATATTTGCAGAATGCATTCGCAGCATTTCTGGCAAATATTTCGTATTAACAAAACCTGTCAGGCCAGGGCTTTTTGCAAAAGGCATTGTGACATATATTTTGTATATGTCAAACTCTAGCTAAAGTTTAAAGTTATTCAAAACTATTTTAGGAAAGTACGGAGAGCAGAATACCATATTTTATTGCTAAAATTTCTTTTTCTTCAGAAACTTAACAAATATCTTATTTCATTTCAGATTTCAATCTTGATATTATAAAAATTTTGCTCTTGCTACAAAATGTTGGATTTTTCTTCAAATAAGTCGGAACAAGGTTACGCCATATTTTCTTCTGTTGTTCCGACAAGCATTGGGACATCGGGCGCACAAAAAAACGACGGCGGGCCCGCCATGCATTCGAAGCAGGGGACGGTCGCTTATAACGTCGCAGCCGGGAATGCACAATATACTGATTTAGGAAAGACCT**CACAAGACGTAATGgtaag**aaataaaccagttaaatattaagttattttttaactgtttggtggctcattttggtatgactgaagttatattaaaattcatacatttttttatattttttaaagatttttttacgatagtttagtttttacaatattttaacggtatgattttacagGATCTCTTCCAAAAAGACACAGTCAACAGTAACAACAACAACAACAATAACGACAAAACAGAAAAGCCAAAAAAGAAGCGCAGACGCGAAAGAAGTCCAGTGACAATTAACAAGTTAAAAAAGATTCGACGATCAAAAGCTAATGACAGAGAACGAAACCGAATGCATGGTCTTAATGACGCACTAGAAGAATTAAGgtaagatgcgaattttttgaacatttttaattaaaatgttatcattaaaaaatttctacagaaaagtagcaaaaagcgttttcaaattattgccaaaatgataaaagaaataatttcaaaagtttaaatcaaaactaaatgatttcttttcttacagACATGTTTTGCCGACCTACCCCGACGAGACCAAATTAACAAAAATCGAAACCTTGCGATTCGCATACAATTATATTTGGTGTTTGAGTGAAATGTTAAAAAATGGAACAGCGGGAAATAGTTTTGgtaagtttgctaaccaatacgttaaagttgatctaccaaaattgtttttttgggggggttagctttttcgtattataaacactttttaaaaattctgaaacatatttttaacattttaattttttaacagATGCAAAATCCGCCGCTCAAGAAATGATGTCATCAGTTCTGACGTCATCTCAAGTAGGCACCGAAAACTTCCCAACCGCGCCCACGGCATTTCAAAACTTCGCCCCCTCCAATGACGTCACAGCTACGTCATACCAGCAGCCTGGGCAATACTACCAGCAATACCCAAACCAGCAACAATATCGACCAAATCGTGACGTCATGCAGCCTACTGTGACGTCACTGGAAATCCCCGACGACCTAGAAAGCATCCAAGCTCCTTTTATGTCGTCACAATCTGACTTTGTTCTGGAGCAAGTGGTCACAAACCAAGCTAACCAAGGAAACGAGATTTTCTTTCAAAATCAACAGATACCAAACAACAATAATAACAACAACAACAGCAACAACAACAACAACAATGTTCAAGCAGTTTTGCCGTCGATTTCGAACTCGTTGTTGGCTAGAAGAAACATCTCGTCTCCAATGACGTTACCCAGCAATGGACAAGTCCAATTTCAGGCCCCCATTTCACCTCCATTTTCTGGGAATTCCCCGACGTGCCCCAGCCCCATGTACCCAGTTGCACAGCCCAACTTTTACATGCCGCCGACCCCAAGCGATTGTGGAAGTATGGCGGGATCGTCGCCCTTCAATAGCCCGCAGAAGCAACTTCCTTCTCCCGATGTACCATACATGCCAAACTACTCCATGCATTTACTCCGATAAGGAAAATCTATAAACATTCCCTATTATGGCTTCAAAACGCCAAAATTCATTCTATTTCATTTGTACGCTCTTTTAGGCGAAATTTTCATTTTCCTTACTGCCTTTTATTACCATTATAAATTGTCCGCCAAACGTATCGTGCCAACTTTCTTTTGAATGTTTATTTCTTACACCTGTGTTCTTAAATAGAACTTCCCACAAACCTaaacttaagtgttttttctacaaaccagtcaaagccaaaaatgtctcaaactcctgtggaagccctcactaaaacgtcactttaggcgctttctgccttccaatctcaaaagcgca

START STOP PAM Neurog.p1 Neurog.p2 **Neurog.1**

>pCESAx

AscI EcoRI BlpI

Tacgtattaattaaggcgcgcc[driver>transgene]gaattccagctgagcgccggtcgctaccattaccagttggtctggtgtcaaaaataataataaccgggcaggccatgtctgcccgtatttcgcgtaaggaaatccattatgtactatttaaaaaacacaaacttttggatgttcggtttattctttttcttttacttttttatcatgggagcctacttcccgtttttcccgatttggctacatgacatcaaccatatcagcaaaagtgatacgggtattatttttgccgctatttctctgttctcgctattattccaaccgctgtttggtctgctttctgacaaactcggaacttgtttattgcagcttataatggttacaaataaagcaatagcatcacaaatttcacaaataaagcatttttttcactgcattctagttgtggtttgtccaaactcatcaatgtatcttatcatgtctggatcgacaaagtcaaagcggccatcagatctgccggtctccctatagtgagtcgtattaatttcgataagccaggttaacctgcattaatgaatcggccaacgcgcggggagaggcggtttgcgtattgggcgctcttccgcttcctcgctcactgactcgctgcgctcggtcgttcggctgcggcgagcggtatcagctcactcaaaggcggtaatacggttatccacagaatcaggggataacgcaggaaagaacatgtgagcaaaaggccagcaaaaggccaggaaccgtaaaaaggccgcgttgctggcgtttttccataggctccgcccccctgacgagcatcacaaaaatcgacgctcaagtcagaggtggcgaaacccgacaggactataaagataccaggcgtttccccctggaagctccctcgtgcgctctcctgttccgaccctgccgcttaccggatacctgtccgcctttctcccttcgggaagcgtggcgctttctcaatgctcacgctgtaggtatctcagttcggtgtaggtcgttcgctccaagctgggctgtgtgcacgaaccccccgttcagcccgaccgctgcgccttatccggtaactatcgtcttgagtccaacccggtaagacacgacttatcgccactggcagcagccactggtaacaggattagcagagcgaggtatgtaggcggtgctacagagttcttgaagtggtggcctaactacggctacactagaaggacagtatttggtatctgcgctctgctgaagccagttaccttcggaaaaagagttggtagctcttgatccggcaaacaaaccaccgctggtagcggtggtttttttgtttgcaagcagcagattacgcgcagaaaaaaaggatctcaagaagatcctttgatcttttctacggggtctgacgctcagtggaacgaaaactcacgttaagggattttggtcatgagattatcaaaaaggatcttcacctagatccttttaaattaaaaatgaagttttaaatcaatctaaagtatatatgagtaaacttggtctgacagttaccaatgcttaatcagtgaggcacctatctcagcgatctgtctatttcgttcatccatagttgcctgactccccgtcgtgtagataactacgatacgggagggcttaccatctggccccagtgctgcaatgataccgcgagacccacgctcaccggctccagatttatcagcaataaaccagccagccggaagggccgagcgcagaagtggtcctgcaactttatccgcctccatccagtctattaattgttgccgggaagctagagtaagtagttcgccagttaatagtttgcgcaacgttgttgccattgctacaggcatcgtggtgtcacgctcgtcgtttggtatggcttcattcagctccggttcccaacgatcaaggcgagttacatgatcccccatgttgtgcaaaaaagcggttagctccttcggtcctccgatcgttgtcagaagtaagttggccgcagtgttatcactcatggttatggcagcactgcataattctcttactgtcatgccatccgtaagatgcttttctgtgactggtgagtactcaaccaagtcattctgagaatagtgtatgcggcgaccgagttgctcttgcccggcgtcaatacgggataataccgcgccacatagcagaactttaaaagtgctcatcattggaaaacgttcttcggggcgaaaactctcaaggatcttaccgctgttgagatccagttcgatgtaacccactcgtgcacccaactgatcttcagcatcttttactttcaccagcgtttctgggtgagcaaaaacaggaaggcaaaatgccgcaaaaaagggaataagggcgacacggaaatgttgaatactcatactcttcctttttcaatattattgaagcatttatcagggttattgtctcatgagcggatacatatttgaatgtatttagaaaaataaacaaataggggttccgcgcacatttccccgaaaagtgccacctgacgtctaagaaaccattattatcatgacattaacctataaaaataggcgtatcacgaggccct

**Electroporation mixes:**

dnFGR overexpression:

100 µg Neurog[BTN]>dnFGFR (or Neurog[BTN]>lacZ control)

20 µg Neurog[BTN]>H2B::mCherry

70 µg Asic>Unc-76::GFP

Fgf8/17/18 CRISPR:

30 µg Fog>Cas9

10 µg Fog>H2B::mCherry

60 µg U6>Fgf8 sgRNAs (20 µg each) or U6>Control sgRNA

30 µg Asic>H2B::GFP

CA-Mras overexpression (GAD reporter):

70 µg Neurog[BTN]>CA-Mras (or Neurog[BTN]>lacZ control)

20 µg Neurog[BTN]>H2B::mCherry

70 µg GAD>Unc-76::GFP

CA-Mras overexpression (Asic reporter):

70 µg Neurog[BTN]>CA-Mras (or Neurog[BTN]>lacZ control)

40 µg Neurog[BTN]>H2B::mCherry

70 µg Asic>Unc-76::GFP

Neurog overexpression for FACS:

70 µg Neurog -3010/-773+-600>tagRFP/tagBFP

50 µg Neurog -3010/-773+-600stop>Neurog

Neurog:WRPW overexpression for FACS:

70 µg Neurog -3010/-773+-600>tagRFP/tagBFP

50 µg Neurog -3010/-773+-600stop>Neurog::WRPW

LacZ control for FACS:

70 µg Neurog -3010/-773+-600>tagRFP/tagBFP

50 µg Neurog -3010/-773+-600>lacZ

Neurog CRISPR (Asic reporter assay):

30 µg Fog>Cas9

10 µg Fog>H2B::mCherry

60 µg U6>Neurog sgRNAs (20 µg each) or U6>Control sgRNA

70 µg Asic>Unc-76::GFP

Neurog CRISPR (GAD reporter assay):

30 µg Fog>Cas9

10 µg Fog>H2B::mCherry

60 µg U6>Neurog sgRNAs (20 µg each) or U6>Control sgRNA

70 µg GAD>Unc-76::GFP
