# Supplementary material for: Regulation of Neurogenesis by FGF Signaling and Neurogenin in the Invertebrate Chordate Ciona
Source: Front Cell Dev Biol. 2020 Jun 23;8:477. doi: 10.3389/fcell.2020.00477 (PMC7324659; doi:10.3389/fcell.2020.00477)
Supplement: Supplementary file 5 [file Data_Sheet_5.DOCX]

**
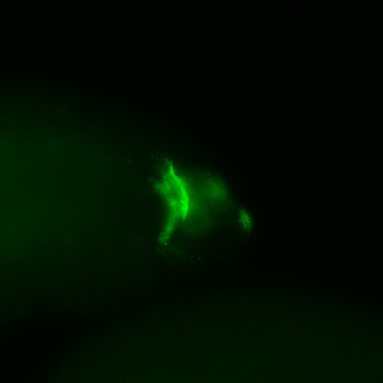
**


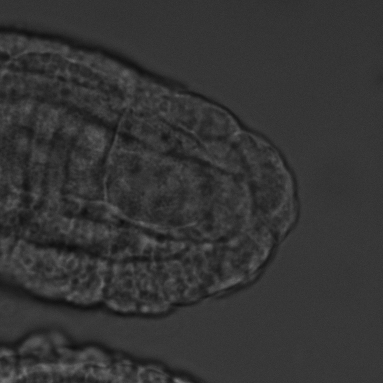

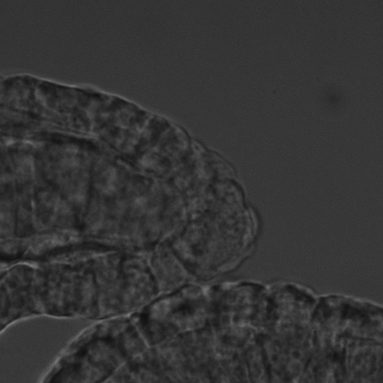

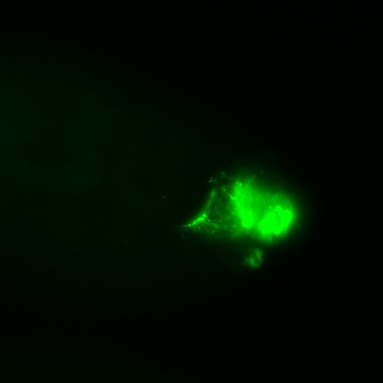


**Supplemental Figure 1. Additional examples of Fgf8/17/18::GFP expression**

More embryos electroporated with *Fgf8/17/18>Fgf8/17/18::GFP* plasmid, showing Fgf8/17/18::GFP (green) emanating from the tail tip cells, spreading around the tip of the notochord. Brightfield panels on left, GFP panels on right. Anterior always to left, dorsal always to top. See figure 2e.


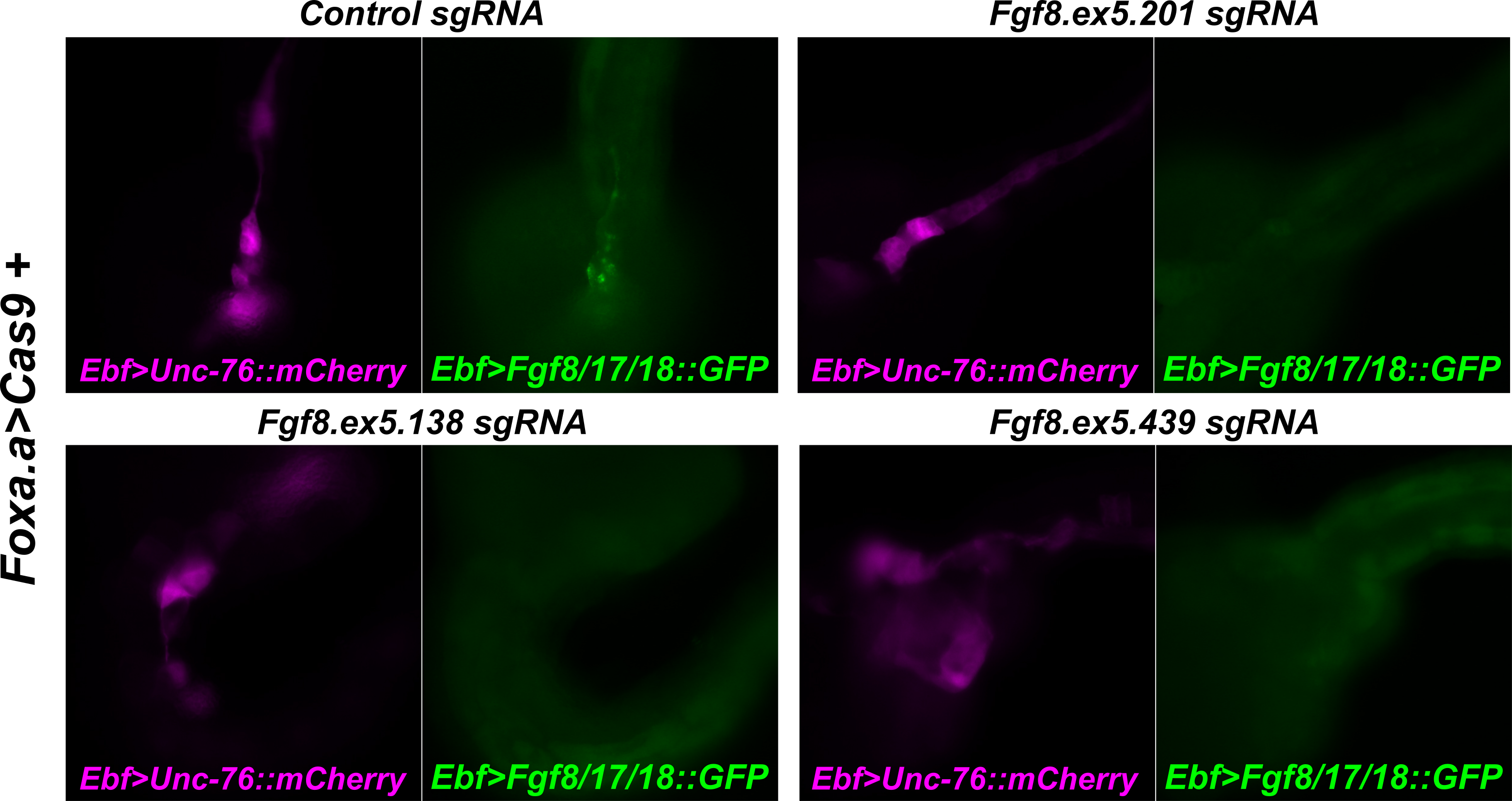


**Supplemental Figure 2. Validation of *Fgf8/17/18* sgRNAs**

Vegetal lineage-specific CRISPR/Cas9-mediated cutting of the *Ebf>Fgf8/17/18::GFP* plasmid was assayed by direct visualization of GFP fluorescence, an sgRNA activity screening technique described previously (Gibboney et al. 2020). All 3 sgRNAs tested resulted in lack of GFP fluorescence in a majority of embryos, though this was not precisely quantified. Compare to control sgRNA (not targeting any endogenous *Ciona* sequence) at top left, which did not result in loss of GFP fluorescence. Plasmids used were: 35 µg *Foxa.a>Cas9*, 70 µg *U6>sgRNA*, 70 µg *Ebf>Fgf8/17/18::GFP,* 50 µg *Ebf>Unc-76::mCherry.*


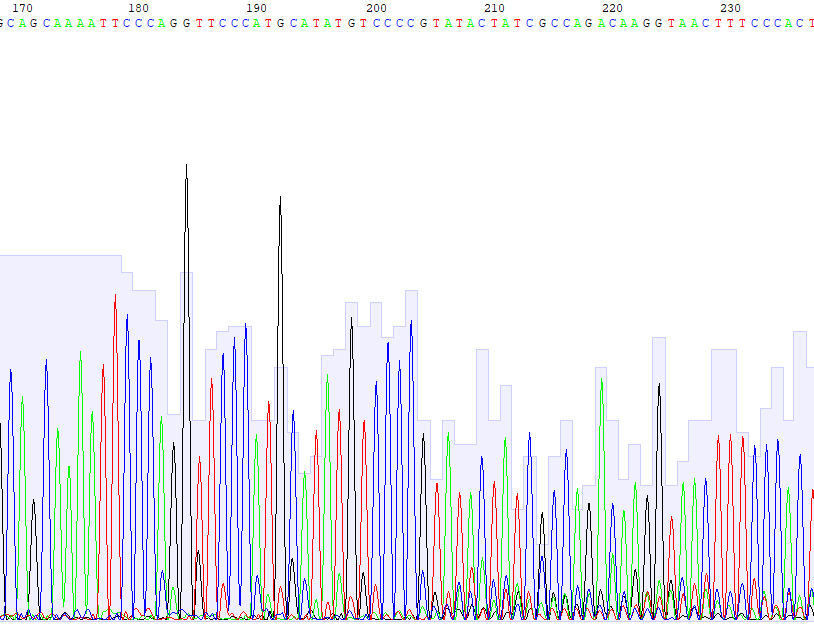


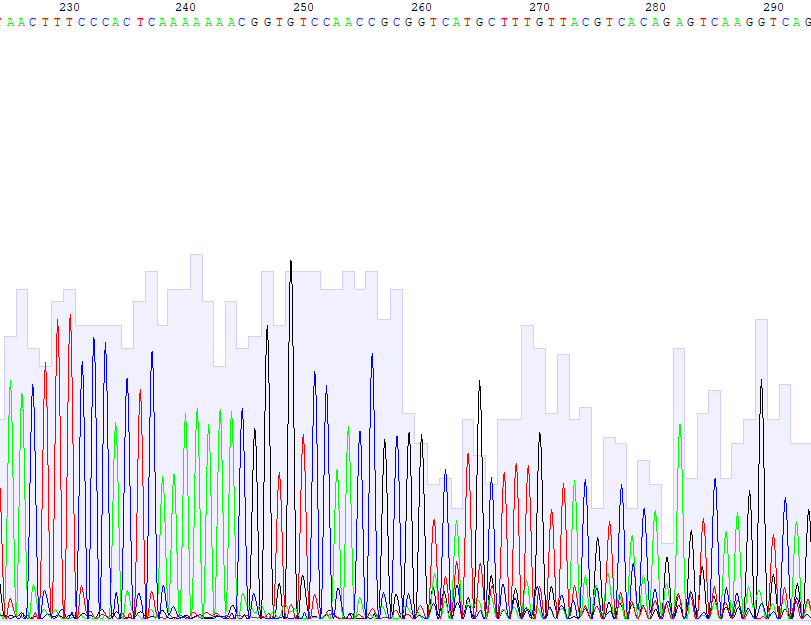


**Supplemental Figure 3. Validation of *Neurog* promoter-targeting sgRNAs**

Sanger sequencing traces of amplicons from embryos electroporated with *Ef1a>Cas9* and *U6>Neurog.p1* (left) and *U6>Neurog.p2* (right)*.* Peakshift scores were calculated to be 0.31 and 0.42, respectively (Gandhi et al. 2018). PAMs indicated by red arrows.
